# Sublexical cues affect degraded speech processing: insights from fMRI

**DOI:** 10.1093/texcom/tgac007

**Published:** 2022-02-16

**Authors:** Arkan Al-Zubaidi, Susann Bräuer, Chris R Holdgraf, Inga M Schepers, Jochem W Rieger

**Affiliations:** Applied Neurocognitive Psychology Lab and Cluster of Excellence Hearing4all, Oldenburg University, Oldenburg, Germany; Research Center Neurosensory Science, Oldenburg University, 26129 Oldenburg, Germany; Applied Neurocognitive Psychology Lab and Cluster of Excellence Hearing4all, Oldenburg University, Oldenburg, Germany; Department of Statistics, UC Berkeley, Berkeley, CA 94720, USA; International Interactive Computing Collaboration; Applied Neurocognitive Psychology Lab and Cluster of Excellence Hearing4all, Oldenburg University, Oldenburg, Germany; Applied Neurocognitive Psychology Lab and Cluster of Excellence Hearing4all, Oldenburg University, Oldenburg, Germany; Research Center Neurosensory Science, Oldenburg University, 26129 Oldenburg, Germany

**Keywords:** superior temporal cortex, sublexical cues, primary auditory cortex, auditory priming, angular gyrus

## Abstract

In natural listening situations, speech perception is often impaired by degraded speech sounds arriving at the ear. Contextual speech information can improve the perception of degraded speech and modify neuronal responses elicited by degraded speech. However, most studies on context effects on neural responses to degraded speech confounded lexico-semantic and sublexical cues. Here, we used fMRI to investigate how prior sublexical speech (e.g. pseudowords cues) affects neural responses to degraded sublexical speech and hence its processing and recognition. Each trial consisted of three consecutively presented pseudowords, of which the first and third were identical and degraded. The second pseudoword was always presented in clear form and either matched or did not match the degraded pseudowords. Improved speech processing through sublexical processing was associated with BOLD activation increases in frontal, temporal, and parietal regions, including the primary auditory cortex (PAC), posterior superior temporal cortex, angular gyrus, supramarginal gyrus, middle temporal cortex, and somato-motor cortex. These brain regions are part of a speech processing network and are involved in lexico-semantic processing. To further investigate the adaptive changes in PAC, we conducted a bilateral region of interest analysis on PAC subregions. PAC ROIs showed bilaterally increased activation in the match condition compared with the mismatch condition. Our results show that the perception of unintelligible degraded speech is improved and the neuronal population response is enhanced after exposure to intact sublexical cues. Furthermore, our findings indicate that the processing of clear meaningless sublexical speech preceding degraded speech could enhance the activity in the brain regions that belong to the cortical speech processing network previously reported in studies investigating lexico-semantic speech.

## Introduction

Integration of speech-specific contextual information can improve speech perception under adverse listening conditions such as noisy environments, particularly for hearing-impaired listeners. Previous studies on the effects of context on speech intelligibility under adverse listening conditions mostly focused on the enhancement of speech intelligibility and neuronal responses of degraded speech by preceding lexico-semantic cues (Obleser and Kotz 2010; Guediche et al. 2016; Holdgraf et al. 2016; Tuennerhoff and Noppeney 2016). However, successful natural speech perception depends not only on lexico-semantic processing but also on sublexical processing (Norris et al. 2000; Dial and Martin 2017). Currently, it is not well understood how sublexical cues affect intelligibility and neural responses to degraded sublexical speech. If sublexical cues could enhance the intelligibility of degraded sublexical speech, then they could also play a role in the enhancement of degraded lexico-semantic speech. Here, we define sublexical speech as the components that make up a proper word (e.g. pseudoword) in a language but are not part of a person’s lexicon (Wendt 2010).

Behavioral evidence for the beneficial effects of sublexical cues for speech perception comes from research with people suffering from hearing loss or language disorders. For instance, Ferguson et al. (2014) reported an improvement in self-reported hearing abilities in patients diagnosed with mild hearing loss after phoneme discrimination training, i.e. training to distinguish between sublexical structures, such as vowels and consonants. Moreover, children suffering from developmental language disorders such as dyslexia showed altered sublexical processing (Bogliotti et al. 2008) and equally benefited from sublexical and lexico-semantic interventions (Wright et al. 2011). Besides, several behavioral studies reported that sublexical (phonemic) cues could facilitate the perception of different forms of degraded speech (e.g. through perceptual learning). For instance, Hervais-Adelman et al. (2011, 2012) used a clear-then-distorted paradigm (presentation of single words or nonwords) to demonstrate that phonological processing could drive perceptual learning and enhanced perception of different kinds of degraded words/nonwords. Likewise, Pallier et al. (1998) and Sebastián-Gallés et al. (2000) reported how nonword and/or foreign language stimuli (i.e. stimuli that lack semantic/lexical cues) could enhance perception and learning of degraded (time-compressed) speech. Recent studies investigating speech perception in aphasic patients found that processing of lexico-semantic information depends on the processing of sublexical cues. These aphasia studies used different tasks, behavioral measures, and eye-movement data (Dial and Martin 2017; Dial et al. 2019).

Task-based fMRI has been used to investigate neural activity in auditory cortical regions in responses to speech stimuli (Formisano et al. 2008; Rutten et al. 2019). Prior neurophysiological studies investigated the effects of lexico-semantic information on speech intelligibility and pointed to a distributed fronto-temporo-parietal network comprising the primary auditory cortex (PAC), posterior temporal cortex (superior temporal gyrus [STG] and sulcus [STS]), middle temporal gyrus (MTG), inferior temporal gyrus (ITG), inferior parietal cortex (angular gyrus [AG], supramarginal gyrus [SMG]), and inferior frontal gyrus (IFG; Lau et al. 2008; Adank and Devlin 2010; Hervais-Adelman et al. 2012; Sohoglu et al. 2012; Davis 2016; Guediche et al. 2016; Holdgraf et al. 2016; Tuennerhoff and Noppeney 2016; Di Liberto et al. 2018).

Lexico-semantic speech context affects speech processing already at the level of PAC (Wild et al. 2012; Di Liberto et al. 2018). In the fMRI study by Wild et al. (2012), matching text enhanced BOLD responses to degraded sentences in PAC compared with degraded sentences paired with mismatching text, indicating PAC modulation by the semantic level within the speech. Such activation changes may be due to rapid tuning shifts in neurons in auditory cortices. In direct electrophysiological recordings from the human temporal cortex, Holdgraf et al. (2016) showed that presenting clear matching versions of lexico-semantic speech before degradation had several effects: it increased speech intelligibility, enhanced neuronal responses, and induced rapid tuning shifts in neuronal population spectrotemporal receptive fields toward task-relevant speech features. In both studies, however, the modulation could have been due to either lexico-semantic or sublexical information, or both.

In posterior middle temporal areas (STG, STS, MTG), Tuennerhoff and Noppeney (2016) found stronger BOLD activation when unintelligible, degraded speech was rendered intelligible by priming the subjects with an intelligible lexico-semantic version of the sentences. This finding is in line with previous studies that observed a positive correlation between speech intelligibility and MTG and STG/STS activation (Obleser et al. 2007; Davis et al. 2011; McGettigan et al. 2012).

AG has been related to successful speech comprehension (Obleser and Kotz 2010; Abrams et al. 2013) and effects of lexico-semantic context integration during degraded speech perception have also been found in AG and SMG (Golestani et al. 2013; Guediche et al. 2016). Using a speech-in-noise paradigm, in which the addition of noise varied semantic predictability of speech, Golestani et al. (2013) found that the BOLD activation in AG, an area involved in speech working memory, increased with increasing intelligibility of contextual speech.

However, the perception of natural speech not only depends on lexico-semantic processing but also on more basic sublexical processing such as the extraction of spectrotemporal features, phonetic features, and temporal structure (Dial and Martin 2017). For example, tuning shifts in PAC have been demonstrated in an animal study. Using simple up/down tone sequences, Yin et al. (2014) observed reward-dependent rapid adaptive changes of spectrotemporal sensitivity in the PAC of ferrets providing evidence for task-dependent short-term plasticity at the earliest stage of cortical auditory processing with nonspeech stimuli. The STG/STS have been implicated in categorical phoneme representation (Chang et al. 2010), speech-specific spectrotemporal processing of nonlexical speech, sentences, and phonetic feature encoding (Mesgarani et al. 2014; Overath et al. 2015; Hullett et al. 2016). Thus, sublexical cues available in lexico-semantic speech may have contributed to the reported enhancement of speech intelligibility and neuronal responses. Therefore, the effects observed in these studies may well have been elicited by sublexical and lexical/semantic processing. Studies mentioned in this paragraph suggest that sublexical stimuli can also drive neuronal activity in the fronto-temporo-parietal network responsive to lexico-semantic speech.

Moreover, the AG also seems to play a role in phonological working memory in addition to speech comprehension (McGettigan et al. 2011; Newhart et al. 2012). This could indicate a role in integrating clear and degraded speech information, independent of semantic content. McGettigan et al. (2011) presented sublexical stimuli to their participants. In their fMRI study, an increase in right AG activation to pseudowords compared with tones was positively correlated with subsequent repetition accuracy. The SMG has similarly been linked to phonological working memory (Paulesu et al. 1993; Henson et al. 2000; Oberhuber et al. 2016) and a recent cortical stimulation study suggests that AG/SMG are involved in storing order information of sensory input in working memory (Papagno et al. 2017).

Taken together, the effects of sublexical cues and lexico-semantic information on the processing of degraded speech could not be isolated in previous neuroimaging studies. Thus, some of the brain areas activated in lexico-semantic processing may also be involved in sublexical processing. In order to investigate brain activity changes in responses to sublexical processing in the absence of lexico-semantic information, we conducted an fMRI study in which identical degraded pseudowords were presented before and after a clear pseudoword. The clear pseudoword matched the degraded pseudowords on *match* trials and did not match the degraded pseudowords on *mismatch* trials. Participants repeated the degraded pseudowords within the scanner and these utterances were recorded to obtain a measure of speech intelligibility. In the match compared with the mismatch conditions, we expected for the second degraded pseudoword (i) improved speech intelligibility, (ii) enhanced neural activation in a fronto-temporo-parietal network that has been previously shown to be activated in successful lexico-semantic processing (Golestani et al. 2013; Clos et al. 2014; Tuennerhoff and Noppeney 2016), and (iii) enhanced PAC responses (Wild et al. 2012).

## Materials and methods

### Participants

Twenty-five right-handed native German speakers with self-reported normal hearing and no history of neurological disorders participated in the study and received monetary compensation. Written informed consent was provided by each participant prior to the experiment. The experiment was approved by the ethical committee of the University of Oldenburg. Five participants were excluded from the analysis. Two participants always repeated the clear pseudoword and/or a common word (e.g. nicht verstanden, German for “not understood”) after the second degraded pseudoword had been presented. The other 3 participants did not complete the experiment. The final data analysis was therefore performed on 20 participants (11 male, mean age = 24.6 years, SD = 3.76 years).

### Stimulus material

The auditory stimulus material consisted of 204 degraded and clear 2-syllabic pseudowords taken from the “Magdeburger Prosody Corpus” (Wendt 2010). All presented pseudowords were phonetically balanced and selected after phonetic and phonotactic rules specific to the German language. However, none of the pseudowords were part of the German vocabulary and had a meaning in German. All audio files had a sampling frequency of 44.1 kHz and were normalized to the same root mean square amplitude.

The speech intelligibility of the stimulus material was reduced, i.e. degraded, by applying a modulation filtering procedure and suppressing some of the power modulations relevant to physical characteristics of speech (Elliott and Theunissen 2009; Santoro et al. 2014). For every single pseudoword, a degraded pseudoword (DW) was created with the following approach (Fig. S3, see online supplementary material for a color version of this figure): First, the log-amplitude spectrogram of the 1D sound file was calculated. Second, the 2D Fourier transform to the original log-amplitude spectrogram was performed to obtain the modulation power spectrum (MPS). Third, to filter out some speech-related modulations, we set all coefficients with power 70–75% of the maximum power to 0. This power range represents a trade-off between severe stimulus degradation and maintenance of essential speech characteristics, such as formants, which are represented by low spectral modulation frequencies centered in the core of the MPS (Elliott and Theunissen 2009). Finally, the modified MPS was back-transformed to obtain DWs by a spectrum inversion algorithm that converts it back into a 1D waveform (Griffin and Lim 1984).

### Experimental design

We employed an auditory priming paradigm to investigate the effect of clear sublexical speech on the perception and neural activation of subsequent degraded sublexical speech. We presented a sequence of three pseudowords on each experimental trial: the first (DW1) and third (DW2) pseudowords were identical and degraded. The second pseudoword was always presented in clear (CW) form and either matched (match condition) or did not match (mismatch condition) the degraded pseudowords with equal probability. The stimuli were presented over in-ear headphones (Sensimetrics Model S14) and the MRI background noise was reduced by damping headphones. Participants were instructed to fixate a cross at the center of the presentation screen and to pay attention to each pseudoword in a trial during the whole experiment.

**Fig. 1:**
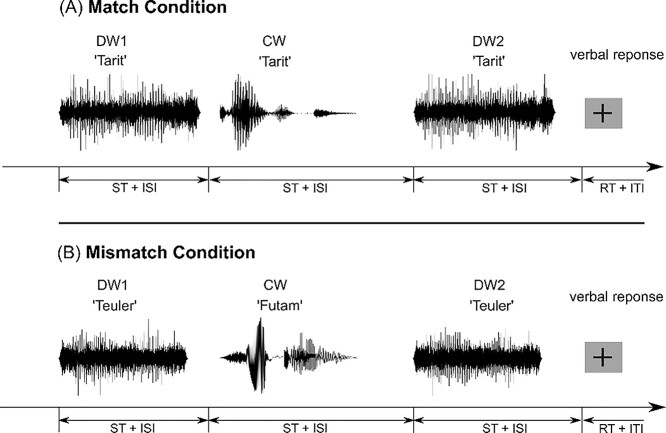
Trial structure in match and mismatch condition. a) Match and b) mismatch conditions consisted of 3 successively presented pseudowords. The first and last pseudowords were degraded (DW1, DW2) and the intermediate pseudoword was presented in a clear form (CW). In the match condition, all 3 stimuli represented the same pseudoword. In the mismatch condition, the clear intermediate pseudoword differed from the degraded pseudowords. Stimulus time indicates the pseudoword length. After the visual cue, participants reported DW2 into a microphone and then pressed a button (RT = response time) to start the next trial. Note that the duration of all interstimulus and intertrial intervals (ISI and ITI) was jittered.

Participants were instructed to verbally report the degraded pseudoword in each trial as soon as the fixation cross changed its color from gray to green after a predefined interstimulus interval with temporal jitter (ISI; Fig. 1). These responses were recorded with an MRI-compatible fiber-optic microphone (Model FOM1-MR, Micro Optics Technologies, Inc.). After the response, participants initiated the subsequent trial via a button press. Each pseudoword was only presented in one single trial and no feedback was given on whether the pronunciation was correct. Therefore, participants could not rely on their vocabulary or learn new pseudowords over trials. To ensure that participants paid attention to all 3 pseudowords, we included catch trials in which the last degraded pseudoword was missing and participants had to verbally repeat the degraded pseudoword based on the first presentation. Catch trials were also divided into match and mismatch conditions. In total, 61 matches, 61 mismatches, and 12 catch trials were presented in a pseudorandom order in four experimental runs of 20 min length, with 5 dummy scans at the start of each run. Intertrial intervals (ITI) and ISIs (Fig. 1) were drawn from a gamma probability density distribution with a minimum of 3 s and a maximum of 16-s length to efficiently sample the consecutive BOLD responses (Boynton et al. 1996). Before scanning, all participants performed a training block with 6 trials inside the scanner.

Functional *T*_2_*-weighted BOLD contrast images were continuously sampled on a 3 T SiemensVerio MR-scanner in sequential ascending order (scan parameter: echo time = 25 ms; repetition time (TR) = 2000 ms; flip angle = 80°; voxel resolution = 3 × 3 × 3.1 mm; field of view = 190 mm, 38 slices). One additional *T*_1_-weighted high-resolution anatomical scan was acquired with an MPRAGE sequence for each participant (scan parameters: TR = 1900 ms; voxel resolution = 1 × 1 × 1 mm; FOV = 256 mm).

### FMRI data preprocessing for whole-brain analysis

The BOLD-imaging data were preprocessed using Statistical Parametric Mapping (SPM12, Welcome Center for Neuroimaging, London, UK) and Matlab (Version R2017b). Preprocessing of the functional images was performed in the following sequence: (i) Head motion correction was achieved by realigning all functional images with one reference image. All 4 runs were first realigned to each other, by aligning the first image from each run to the first image of the first run. Then, the images within each run were aligned to the first image of the run. There were two steps for realignment. In the first step, a rigid-body (3 translation parameters, 3 rotation parameters) transform was determined between each individual image and one reference image from the same participant by using a least-squares approach (standard SPM algorithm). In the second step, the parameters were applied to the functional images to reslice all images using the seventh degree B-spline interpolation. (ii) Temporal differences caused by slice time acquisition were corrected by applying a Fourier phase shift interpolation (standard SPM algorithm). The middle slice was used as a reference slice. (iii) The functional and *T*_1_-structural images were coregistered to the mean functional image. (iv) The parameters from the segmentation of the *T*_1_-structural image were used to normalize the functional images to the Montreal Neurological Institute (MNI 152) template. (v) A Gaussian kernel with a full-width at half maximum of 8 mm for spatial smoothing was applied. (vi) A high-pass filter with a cut-off at 1/128 s was used to remove potential low-frequency signal drifts.

### Statistical analysis

#### Behavioral performance

To obtain a measure for speech intelligibility, the behavioral results were quantified as the percentage of a scored verbal repetition performance. This measure was based on a whole-pseudoword recognition score with the grading of 1 (a pseudoword identified without mistake), 0.5 (a pseudoword defined with only one incorrectly reported phoneme), and 0 (more than one phoneme was incorrectly reported). To reveal differences between the match and mismatch condition, a paired *t*-test was performed across participants.

In addition, we report the percentage of correct and incorrect responses of subjects on catch trials and the clear pseudoword repetition in mismatch conditions. For details, please see the supplementary material (Figs. S1 and S2, see online supplementary material for a color version of this figure).

#### Sound envelope correlation

To compare the shape of the envelopes between the filtered and clear pseudoword in each match trial, the pseudowords’ wideband envelopes and the Pearson correlation between the envelopes were calculated (Gross et al. 2013). In short, we band-pass filtered the speech signal in 9 logarithmically spaced frequency bands between 100 and 10,000 Hz, similar to the cochlear frequency map. Next, the filtered time series were Hilbert transformed and the absolute values for each band were summed to obtain the wideband envelope of the acoustic signal.

#### Whole-brain analysis

Statistical analysis of the fMRI data was performed using the general linear model (GLM) implemented in SPM12 (Wellcome Center for Neuroimaging, London, UK) and Matlab (Version R2017b, MathWorks, Inc., Natick, MA, USA). Each voxel time course was modeled as a linear sum of BOLD activations induced by the variations of the experimental variables. We included the onsets of 8 events of interest in this model: the onsets of the 3 pseudowords presented in each trial, separately for each condition (match, mismatch) plus the onsets of the verbal responses for the match and the mismatch conditions. These onsets were then convolved with the standard hemodynamic response function in SPM to obtain the model activation time-courses and used as regressors in a fixed-effects GLM analysis. In addition, we added the movement parameters from the realignment step as variables of no interest to the model. For each subject, individual contrast images were computed for each GLM regressor against the baseline to be used in statistical group-level analyses. The group-level analysis consisted of 2 separate repeated-measures analyses of variance (rm-ANOVA). The first rm-ANOVA with 1-factor “pseudoword type” had 3 levels, the two measurements of degraded pseudowords (DW1, DW2) and one measurement of a clear pseudoword (CW). This analysis was employed to identify BOLD activations elicited by degraded and clear pseudowords irrespective of the factor match/mismatch. Between 3 levels of 1-factor rm-ANOVA, we performed a set of whole-brain conjunction analyses (CAs) to test for overlaps in BOLD effects across the pseudoword types, again irrespective of the condition. The CA combined the *F*-statistic maps for each pair of pseudowords (DW1/DW2, DW2/CW, and DW1/CW) and the pseudoword triple (DW1, CW, and DW2) separately. For each CA, we applied the conjunction-null-hypothesis approach, as implemented in SPM12 (Friston et al. 2005; Nichols et al. 2005). We then performed a second rm-ANOVA to test the 2-way interaction between the two factors: position of DWs (2 levels: DW1, DW2) and condition of clear pseudoword (2 levels: match, mismatch). All statistical significance results were reported from the F-tests at *P* < 0.05 FWEc (family-wise error cluster level) with an initial threshold at the voxel level of *P* < 0.001 (uncorrected) to define a cluster threshold (k). Significant clusters were anatomically labeled according to the AAL atlas (Tzourio-Mazoyer et al. 2002) included in the xjView toolbox (http://www.alivelearn.net/xjview8).

In addition to the analysis concerning trial-type, we also performed an analysis concerning response-type (correct or incorrect). Therefore, we reran the subject level analysis by including the onsets of the 3 pseudowords in each trial into the GLM but now according to the behavioral response to DW2 (correct, incorrect) and the onsets of the verbal responses. Again, we computed individual contrast images of GLM regressor against the baseline for each subject. Then, we used the contrast images in statistical group-level analyses. First, to check whether the activation overlap between DW1, CW, and DW2 depended on correct versus incorrect identification of DW2, we performed 2 rm-ANOVAs, 1 for correct and 1 for incorrect responses. Both of them had 1-factor “pseudoword type,” which had 3 levels as described above. These rm-ANOVAs identified BOLD activations elicited by degraded and clear pseudowords with respect to correct and incorrect response-type, respectively. In the end, the CA combined the F-statistic maps for each pair of pseudowords as above. Second, to investigate whether the interaction analyses between the response-type (correct, incorrect) or condition of clear pseudoword (match, mismatch) provided similar results for the BOLD responses of the degraded pseudoword position (DW1, DW2), we extracted the average beta-values of voxels of regions of interest (ROIs) that contribute to the speech processing network using the Harvard-Oxford cortical and subcortical atlas (RID: SCR_001476; Fig. S6a, see online supplementary material for a color version of this figure).

#### PAC ROI analysis

The PAC activity level is associated with the more complex and processing of spectrotemporal acoustic features of lexico-semantic speech (Miller et al. 2002; Theunissen and Elie 2014). Here, we used ROI analysis to investigate whether changes in PAC activity in response to the degraded pseudowords are enhanced by matching prior sublexical speech cues compared with mismatched cues.

For the PAC ROI analysis, we adapted the method applied by Wild et al. (2012). We first divided PAC (Brodmann area 41) into 3 subregions, Te1.0, Te1.1, and Te1.2, using the template provided by the SPM Anatomy toolbox (Eickhoff et al. 2005). These templates are based on the cytoarchitectonic mapping results of Morosan et al. (2001). Next, we extracted the beta-values for the regressors of the first and second degraded pseudowords (DW1, DW2) of the match and mismatch conditions from all voxels in these regions showing activation modulation in any experimental condition (effects of interest *F*-contrast *P* < 0.001, uncorrected) using MarsBaR v0.42 (Brett et al. 2002). This selection criterion mitigates a potential selection bias while excluding voxels that carry only noise. This analysis step was performed on MNI-transformed data without additional spatial smoothing. Then, we averaged the extracted beta-values corresponding to DW1 and DW2 for each subject, condition, and ROI separately. Finally, a 2 × 3 × 2 factorial rm-ANOVA with factors condition of clear pseudoword (match, mismatch), ROI (Te1.0, Te1.1, Te1.2), and hemisphere (left, right) was performed with SPSS (Version 24.0. Armonk, NY: IBM) and post-hoc *t*-tests (false discovery rate [FDR] corrected at *q* = 0.05; Benjamini and Hochberg 1995).

**Fig. 2:**
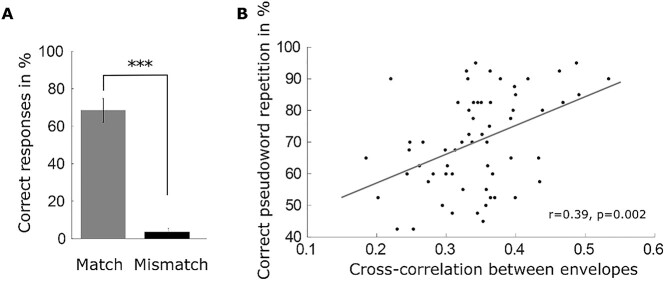
Behavioral results of the speech intelligibility task. a) Matching clear pseudowords increased the intelligibility of the degraded pseudowords significantly in a paired *t*-test (*** represent *P* < 0.001). Bars show the mean with the standard deviation over subjects. b) The correlation between the speech envelopes of degraded and matching clear pseudowords predicted the individual pseudoword intelligibility. Points represent the mean percentage of correct responses over participants for individual pseudowords after hearing the clear and degraded pseudowords.

## Results

### Behavioral results

The paired *t*-test of the correct responses revealed a significant (*t*(19) = 24.84, *P* < 0.001) difference between conditions (match vs mismatch). On average, participants repeated degraded pseudowords more accurately in match trials (*M* = 68.4%, SD = 12.64%) than mismatch trials (*M* = 3.4%, SD = 3.82%; Fig. 2a). A potential factor for the observed increase in correct repetitions might be the similarity between the speech envelopes of degraded and clear pseudowords in match trials. In order to test this hypothesis, we first cross-correlated the speech envelopes of CW and DW pairs in the match condition and extracted the maximum correlation across lags. Across matching pseudowords, the speech envelope correlation ranged between *r* = 0.18, indicating low similarity between the 2 envelope signals, and *r* = 0.53, indicating high similarity. Second, we related envelope similarity with the correct repetition rate for the individual pseudowords using Spearman rank correlation. This analysis revealed a positive correlation between CW-DW envelope correlation and correct pseudoword repetition (*r* = 0.39, *P* = 0.002, Fig. 2b). Furthermore, individual pseudoword intelligibility ranged between 42.5% and 95%. The percentage refers to the proportion of participants who correctly repeated that pseudoword. In sum, a matching CW can enhance the intelligibility of the DW and the correlation between CW and DW envelopes is a good predictor of degraded pseudoword repeatability in the matching CW condition.

In catch trials, the participants repeated DWs more accurately in match trials than mismatch trials (Fig. S1, see online supplementary material for a color version of this figure). Moreover, we analyzed the proportions of repetitions of the CW in the incorrect responses to mismatch trials as an indicator of task adherence. We found that in the incorrect trial, subjects repeated only 13.6% the CW instead of the DW (Fig. S2, see online supplementary material for a color version of this figure). These results support our assumption that the participants tried to attend to DW1 as well as DW2 and that the answers were in the vast majority, not simply a repetition of the CW. As stated in Materials and methods section, we actually excluded 2 participants whose audio recordings of their verbal responses indicated noncompliance with task instructions (see Materials and methods).

### FMRI BOLD activations elicited by clear and degraded pseudowords

**Fig. 3:**
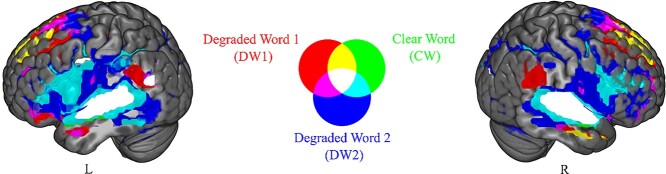
FMRI BOLD activations elicited by clear and degraded pseudowords. Results for the main effects of the degraded pseudoword at the beginning of each trial (DW1, red), the clear intermediate pseudoword (CW, green), and the subsequently degraded pseudoword (DW2, blue). Conjunction results of DW1 and CW (yellow), CW and DW2 (light blue), DW2 and DW1 (magenta), and all pseudoword types (white). All statistical results were assessed for cluster-wise significance using a cluster defining threshold *P* < 0.001 at the voxel level and *P* < 0.05 FWEc corrected at the cluster level.

First, we analyzed which ensembles of brain areas were modulated by each of the 3 pseudowords presented in each trial, irrespective of the factor match/mismatch. Fig. 3 shows the color-coded voxels that significantly changed activity (*P* < 0.05, FWE corrected at cluster level). Red, green, and blue areas represent the main effect of word type: DW1, CW, and DW2, respectively (see Table S1, see online supplementary material, for the number of voxels per cluster and *F*-values). Furthermore, the mixed colors represent conjunctions of activations, i.e. these brain areas were significantly modulated by either 2 or all 3-word types. The colors indicate the conjunction of DW1 and CW (yellow), DW2 and CW (cyan), DW1 and DW2 (magenta), as well as the conjunction of DW1, CW, and DW2 (white). This analysis provides an overview of brain regions responding to one or several types of the 3 pseudowords and visualizes where the BOLD modulations overlap between DW1, DW2, and CW.

According to whole-brain CA, both, degraded and clear, pseudowords elicited overlapping activations along a bilateral ventral speech processing stream consisting of posterior/middle STG, superior temporal sulcus (STS), the temporal plane, left AG, and an additional cluster in the left premotor cortex (Fig. 3, white voxels and Table S1, see online supplementary material). Physically identical degraded pseudowords elicited different activation patterns depending on their position in the trial. In general, DW2 changed the activity of the largest ensemble of brain areas (DW2: blue, cyan, and white voxels), followed by CW (CW: cyan and white voxels). All brain areas activated by CW were activated by DW2 as well, with high overlap in the mentioned areas of the temporal lobe and frontal speech areas (Fig. 3, cyan and white voxels and Table S1, see online supplementary material). Importantly, this overlap in activity modulation between CW and DW2 in these areas was larger than the overlap between CW and DW1 (only white voxels) as DW1 modulated fewer voxels than CW (Table S1, see online supplementary material). However, DW1 modulated activity in bilateral supramarginal and AG (red and white voxels) and shared a modulation cluster with DW2 in the left anterior temporal cluster (magenta voxels). In contrast to DW1, CW and DW2 modulated only a small subregion of the left supramarginal and AG indicated by white voxels.

We checked if the activation overlap between DW1, CW, and DW2 changed when participants correctly reported the DWs versus not. We observed similar overlapping clusters of brain activation for correct and incorrect responses (see Fig. S5, see online supplementary material for a color version of this figure). As shown in Figs. 3 and S5, the results of peak voxel activations (*P* < 0.05, FWE corrected at cluster level) in the analysis with respect to the response-type (correct and incorrect)-related analysis were similar, but with smaller clusters, compared with the conditions of clear pseudoword (match and mismatch)-related analysis.

In sum, DW1 modulated fewer areas than CW and DW2. All areas modulated by DW1 were additionally modulated by CW and/or DW2, except SMG, which was implicated in working memory (Oberhuber et al. 2016; Papagno et al. 2017). CW modulated fewer areas than DW2 and all areas that changed activity by CW were additionally modulated by DW2. The observed brain patterns indicate a modulatory effect of sublexical clear speech on the neural processing of DW2, since the brain pattern elicited by DW2 exceeds the brain pattern produced by DW1. This suggests that the processing of the degraded pseudoword shares more modules of the speech processing network with the clear pseudoword after the clear pseudoword has been presented.

**Fig. 4:**
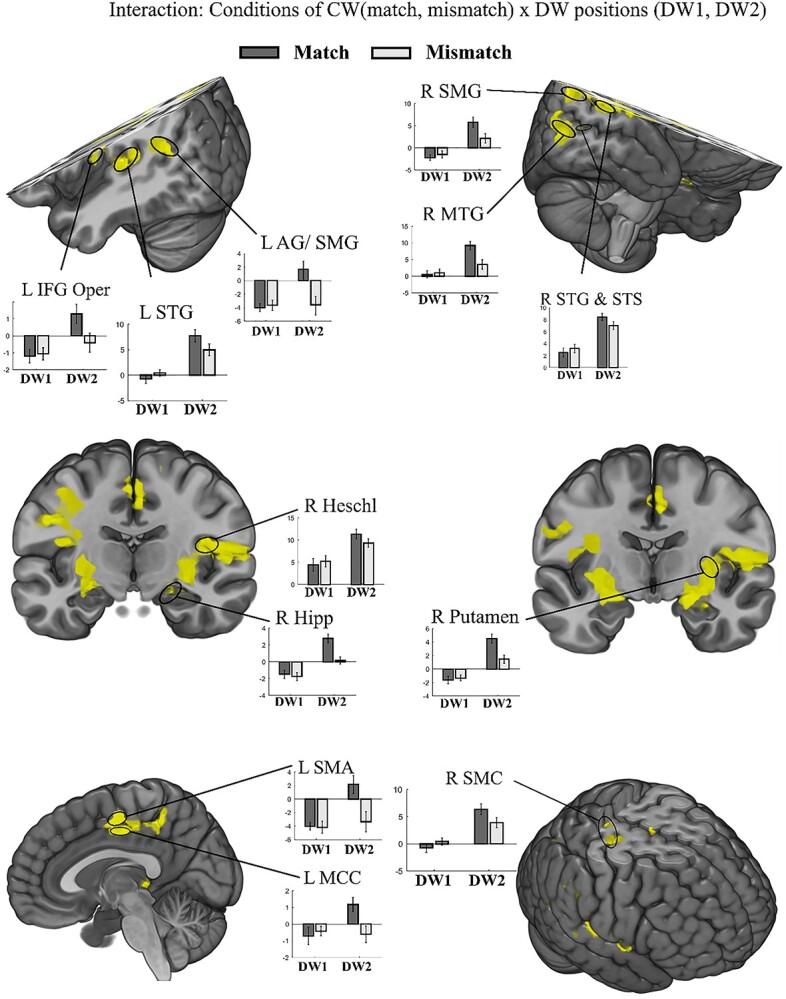
Interaction between the type of clear pseudoword (matching and mismatching conditions) and position of degraded pseudowords. There was a significant interaction of conditions (matching and mismatching) and DWs (DW1 and DW2) in the fronto-temporo-parietal speech network. Statistical results were assessed for cluster-wise significance using a cluster defining threshold *P* < 0.001 at the voxel level and *P* < 0.05 FWEc corrected at the cluster level. The bars represent beta-values. Clusters were anatomically labeled according to the AAL atlas. Note that responses to DW1 were similar across conditions, but responses to DW2 were higher in the match than in the mismatch trials. Abbreviations: Hipp: Hippocampus; IFG Oper: Inferior frontal gyrus pars opercularis.

### Interaction between the type of clear pseudoword (matching and mismatching conditions) and position of degraded pseudowords (DW1 and DW2)

The previous analysis did not differentiate between match and mismatch conditions. However, the CW may affect the DW2 processing differently, depending on whether the CW matched DW2 or not. Such a differential effect could reflect enhanced processing of DW2 after the presentation of a matching CW that resulted in the enhanced intelligibility of DW2. To test this hypothesis, we performed an rm-ANOVA in which we tested for a significant interaction between DWs (2 levels: DW1, DW2) and clear pseudoword conditions (2 levels: match, mismatch). We found significant interactions of BOLD activations between condition (matching or mismatching) and position of degraded pseudowords (DW1 or DW2) in multiple brain areas of the speech processing network (Fig. 4 and Table S2, see online supplementary material; Hickok and Poeppel 2007; Rauschecker and Scott 2009; Friederici 2011; Papagno et al. 2017) and beyond. In order to further analyze the nature of the interactions, we plotted beta values of peak voxels in Fig. 4 and average betas of ROIs defined according to the Harvard-Oxford cortical and subcortical atlas in Fig. S6, see online supplementary material for a color version of this figure. These plots indicate that the observed interactions are generally due to increased BOLD responses in the responses to DW2 in the match compared with the mismatch condition. We observed no difference between conditions for DW1 activations (Figs. 4 and S6, see online supplementary material for a color version of this figure, and Table S3, see online supplementary material). This was to be expected as the trial type was undetermined at the time when DW1 was presented.

**Fig. 5:**
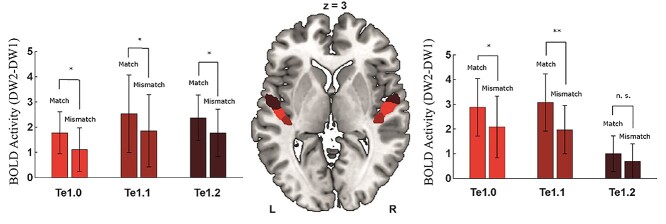
Effects of matching clear pseudowords on degraded pseudoword BOLD activation in PAC ROIs. The three different colors mark the cytoarchitectonically distinct PAC regions on an axial slice (black = Te1.0, white = Te1.1, gray = Te1.2). The left bar graphs show BOLD activations for the match and mismatch condition in the respective ROIs in the left hemisphere and the right bar graphs that show the respective BOLD activations in the right hemisphere; 5 of the 6 PAC ROIs showed greater BOLD activation for the match compared with the mismatch condition in paired *t*-tests (** represent *P* < 0.01, * represent *P* < 0.05, FDR corrected; n.s. = not significant). Bars show the mean with the standard deviation over subjects.

Importantly, in STG, STS, and Heschl gyrus, which are all areas of the speech network (Hickok and Poeppel 2007; Rauschecker and Scott 2009; Friederici 2011; Papagno et al. 2017), the interaction was significant and the BOLD activity elicited by DW2 was higher in the match compared with the mismatch condition (Fig. 4; see Table S2, see online supplementary material for the number of voxels per cluster and *F*-values). This observed activation pattern, together with a significant increase in speech intelligibility for DW2 in the match condition (Fig. 2), may indicate an enhanced representation of important sublexical speech features in the temporal lobe for DW2 induced by the preceding CW. Furthermore, we found the same interaction pattern in left AG and left SMG, 2 areas associated with phonological working memory as well as higher level categorical and syntactical speech processing. Moreover, we found interaction with increased DW2 activation in several voxels in right posterior MTG, right SMG, left posterior supplementary motor area (pSMA), left middle cingulate cortex (MCC), bilateral putamen (an input structure to the basal ganglia), bilateral somato-motor cortex (SMC), and ventral premotor areas in IFG reaching anteriorly to pars opercularis exhibited and interaction (Fig. 4). The bilateral IFG and SMC are known to be recruited under difficult hearing conditions (Eckert et al. 2012; Rampinini et al. 2017; Alain et al. 2018) and their activation level is predictive of memory performance for speech in noise (Vaden et al. 2017), correct syllable processing (Markiewicz and Bohland 2016), and phoneme recognition (Callan et al. 2014). We also found interaction effects in brain areas that contribute to lexico-semantic context processing of speech, such as the right ITG (Fig. S6, see online supplementary material for a color version of this figure, and Table S2, see online supplementary material; Davis 2016; Lau et al. 2008).

Finally, we found similar interaction patterns across ROIs (Fig. S6b, see online supplementary material for a color version of this figure) when we plotted the beta values with respect to the behavioral response to DW2 (correct, incorrect) or conditions of CW (match, mismatch). This similarity was expected as most correct responses were in match trials, while most incorrect responses were in mismatch trials. However, the differences appeared somewhat larger when trials were grouped according to condition. Also, we observed that activations in STG ROIs were a little higher than activation in the Heschl gyrus.

### PAC ROI analysis

Previous analyses were performed on the whole-brain level and did not specifically test for the earliest cortical level at which speech is processed, such as the PAC (Whalen et al. 2006). In the last step, we specifically analyzed the adaptive changes in PAC subregions by comparing the modulatory effect of matching and mismatching CW on DW2 activation in PAC (Te1.0, Te1.1, Te1.2). We performed an ROI-based rm-ANOVA with factors condition (match, mismatch), ROI (Te1.0, Te1.1, Te1.2), and hemisphere (left, right). This analysis was performed to gain a deeper understanding of short-term plasticity in PAC in degraded speech processing. The preceding whole-brain analysis already identified increased relative activation changes in PAC for potentially intelligible DW2 in the match condition compared with unintelligible DW2 in the mismatch condition. Here, we address the question of whether this effect can be observed in all 3 cytoarchitectonic areas and both hemispheres equally. The rm-ANOVA yielded a significant main effect of condition (*F*(1,19) = 16.8, *P* < 0.001), showing higher relative activation for the match condition (*M* = 2.27, SD = 0.41) than for the mismatch condition (*M* = 1.58, SD = 0.42). Post-hoc paired *t*-tests revealed that all PAC ROIs, except right Te1.2, showed greater activation in the match condition than the mismatch condition (*P* < 0.05, corrected; Fig. 5). There was no significant main effect for the hemisphere and ROI and there were no interaction effects with the factor condition. To test whether the standard deviation (std) of beta-values across voxels in ROIs between conditions could have also changed, we recomputed the rm-ANOVA by including the std instead of the mean beta-values. We found no indication for a change in the standard deviation of beta-values across ROIs (see Fig. S4, see online supplementary material for a color version of this figure).

## Discussion

In the present study, we investigated the neuroanatomical activation changes associated with improved speech perception of degraded sublexical speech through matching clear sublexical speech. The major difference of our study compared with previous studies is the absence of lexico-semantic information. Our findings demonstrate that the presence of clear sublexical speech, independent of lexico-semantic information, is sufficient to overcome ambiguities in degraded sublexical speech and enhances cortical responses in a broad fronto-temporo-parietal speech network that has been reported in lexico-semantic speech processing. In many areas of the speech processing network, this effect shows specificity to the speech context with matched intact and degraded pseudowords having stronger effects than unmatched. Moreover, our results further support the notion that informative contextual sublexical cues can trigger rapid adaptive changes in the PAC that could support the processing of degraded sublexical speech and help improve intelligibility.

### Context effects on intelligibility

In line with previous studies using lexico-semantic stimuli, we demonstrate at the behavioral level a significant improvement in degraded pseudoword perception after the presentation of a matching clear pseudoword compared to a mismatching one (Sohoglu et al. 2012; Clos et al. 2014; Holdgraf et al. 2016). In contrast to these studies, we demonstrate this improved intelligibility for the purely sublexical speech by using pseudowords and identifying the remaining envelope information as a predictor of successful speech comprehension (Drullman 1995). It is well known that envelope information is important for speech intelligibility (Shannon et al. 1995). Furthermore, both auditory and visual speech envelope information is represented in cortical neural responses (Micheli et al. 2020). Our results suggest that sublexical speech cues are beneficial for the intelligibility of the degraded pseudoword when their temporal speech structure is preserved and similar to the clear pseudoword. Thus, the success of sublexical cues processing may at least partly be mediated by the remaining envelope information still present in the degraded pseudoword.

### FMRI BOLD activations elicited by clear and degraded pseudowords

We hypothesized that successful sublexical speech processing of degraded speech would increase brain activation in core areas of the speech network, although pseudowords are unlikely to activate a semantic processing system. Therefore, at the whole-brain level, we investigated whether the activity of fronto-temporo-parietal speech processing network was modulated by sublexical cues or not. We found that the 3 types of pseudowords (DW1, CW, DW2) elicited overlapping activations in bilateral posterior and middle STG/STS and the temporal plane. These areas are commonly activated by speech and nonspeech auditory stimuli in fMRI (Price 2012). More importantly, DW1 and DW2 also showed differences in the neuroanatomical extent of their activation patterns. DW2 led to a wider activation in STG and STS compared with DW1. Primarily, we found a more extensive activation overlap between CW and DW2 than between CW and DW1, providing the first hint that prior CW presentation improved speech processing of DW2. Since identical stimuli were used as DW1 and DW2, the difference in activation is determined by the position of the degraded pseudoword in a trial, before or after the CW, and not by the physical properties of the stimulus. However, this analysis did not test for the differentiate between the effects of presenting a matching or a nonmatching pseudoword. Therefore, the observed effects may have been unspecific to the information provided by the clear pseudoword.

### Interaction between the type of clear pseudoword (matching and mismatching conditions) and position of degraded pseudowords (DW1 and DW2)

We performed an additional interaction analysis to probe the specificity of the activations for the successful processing of sublexical cues in the degraded speech. To demonstrate this specificity, we calculated a match/mismatch interaction contrast as the behavioral results showed that only the matching pseudowords are successfully integrated into the processing of degraded speech.

As a first important observation and proof of the quality of the data presented, pseudowords drove the activation within expected brain areas of the speech processing network (Hickok and Poeppel 2007; Rauschecker and Scott 2009; Friederici 2011; Papagno et al. 2017), including bilateral activation of the superior and middle temporal lobe, the inferior parietal cortex, and inferior frontal brain areas. Notably, the activation extent depends on the type of presentation (degraded or clear) and position of the degraded pseudoword in a trial.

We found a similar pattern of brain activation across ROIs (Fig. S6b, see online supplementary material for a color version of this figure) when analyzing the data according to the behavioral response to DW2 (correct, incorrect) or condition of CW (match, mismatch). This similarity was expected as most correct responses were in match trials while most incorrect responses were in mismatch trials. Note that, we did not have confidence trials in our experiment to judge if the participants’ responses were totally confident or not (Rieger et al. 2008). However, our study aims to demonstrate that sublexical cues affect the brain regions related to speech processing and was not designed to analyze the effects of correct/incorrect behavioral responses. Therefore, we discuss the BOLD activation results of DW2 concerning the grouping by match versus mismatch conditions below.

The match conditions showed increased activation for DW2, particularly in temporal and parietal areas, including the STG/STS, MTG, AG, SMG, SMC, and pSMA. However, this effect was weaker or absent in the mismatch condition, indicating the specificity of the matching clear pseudoword and causing the interaction. One interpretation suggested by the results of Holdgraf et al. (2016) is that the neural representations of degraded pseudowords (DW2) are enhanced, possibly by tuning shifts of the neuronal populations toward the acoustic features of the clear pseudowords after hearing the clear (unfiltered) pseudoword. Such response alterations indexing short-term plasticity were reported for lexico-semantic speech in electrophysiological and neuroimaging studies (Wild et al. 2012; Hakonen et al. 2016; Holdgraf et al. 2016; Sohoglu and Davis 2016).

In the current findings, the increased STG/STS activation in the match conditions may reflect enhanced processing of spectrotemporal speech features. Support for this interpretation comes from a recent electrocorticography study. Using an encoding modeling approach (Holdgraf et al. 2017) to derive spectrotemporal receptive fields of intracranial electrodes Holdgraf et al. (2016), the authors showed that previous exposure to intact sentences shifts spectrotemporal tuning of neuronal population responses in STG during the presentation of the same degraded sentences toward the tuning observed during the intact sentence along with improved comprehension. Importantly, in our current study, the activation enhancement shift was induced by sublexical speech features, which is in line with tuning shifts for low-level speech features suggested by Holdgraf et al. (2016). The posterior portions of the STG/STS have been functionally related to categorical phonetic encoding (Chang et al. 2010; Turkeltaub and Branch Coslett 2010) and increased STG/STS and MTG activations have been shown to correlate with speech intelligibility (Obleser et al. 2007; Davis et al. 2011; McGettigan et al. 2011). Taken together, our results are in concordance with the notion that acoustic sublexical speech cues can induce short-term adaptation of the sensitivity profiles to relevant speech features in bilateral STG/STS and MTG to support the successful perception of degraded speech.

Furthermore, match conditions showed a robust activation in bilateral posterior IFG for degraded speech processing (DW2), which is in line with previous research. Clos et al. (2014) presented degraded sentences after matching or mismatching clear and degraded sentences. They observed a relative increase of activation in the left IFG (premotor and pars opercularis) for degraded sentences that were primed with matching clear sentences. They attributed this left IFG activation to the combination of meaningful speech information from the intact sentences in the processing of the degraded sentences. However, as we presented clear sublexical pseudowords, the integration of lexico-semantic context cannot have driven the observed IFG activation increase. The IFG has been associated with phonological encoding (Papoutsi et al. 2009), word-level processing, including sequencing of sublexical elements (Uddén and Bahlmann 2012), and syntax complexity (Friederici et al. 2011). Additionally, there exists a considerable amount of literature demonstrating that IFG is involved not only in speech-related processing but, more generally, also in the spatial and temporal segmentation in different modalities (Maess et al. 2001; Fadiga et al. 2009; Reichert et al. 2014). In light of the wide range of cognitive functions supported by IFG, we surmise that increased IFG activation reflects the processing of sequential sublexical cues in our study. This conjecture is further corroborated by our result that sound envelope shapes predict to some extent the successful pseudoword repetition. This puts our findings in line with a more general functional role of the left IFG in the segmentation and comparison of acoustical segments for behavioral decisions (Bohland and Guenther 2006) than a semantic integration (Clos et al. 2014).

The inferior parietal cortex, specifically the AG and SMG, is activated in tasks involving semantic integration or phonological working memory (Paulesu et al. 1993; Seghier 2013; Oberhuber et al. 2016). The AG and SMG activation in the match conditions (after matching the clear and degrades words, and not in mismatch condition) and large activation during clear pseudoword (irrespective of match and mismatch conditions; Fig. 3) in our study support the notion that they function as integration areas and phonological short-term memory storage, which facilitates speech perception (Jacquemot and Scott 2006). A previous fMRI study showed that bilateral AG could discriminate between intelligible and unintelligible speech, further supporting an important role for AG in successful speech comprehension (Abrams et al. 2013).

We observed a strong activation in SMC and pSMA for the match conditions. Successful perception of degraded speech in the match condition was accompanied by greater SMC and SMA responses than the unsuccessful perception of degraded speech in the mismatch condition. In line with our findings, numerous studies report motor activation while participants listen to speech (Cheung et al. 2016; Jones and Schnupp 2021). For instance, posterior SMA has been shown to be activated in verbal working memory and top-down predictive mechanisms during speech perception (Tourville and Guenther 2011; Hertrich et al. 2016). SMC was postulated to play a supportive role in effortful speech perception but is not required under ideal listening conditions (Davis and Johnsrude 2003; Pulvermüller et al. 2005; Osnes et al. 2011; Hervais-Adelman et al. 2012). However, the exact functional meaning of SMA and SMC activation is unclear. For instance, neuroimaging studies using a passive speech perception and active speech production task showed that the SMC is sensitive to syllable identity, independent of auditory spectrotemporal features of the speech input (Evans and Davis 2015). A study by Cheung et al. (2016) demonstrated that the enhanced activation of ventral SMC reflects sensitivity to precise auditory features independent of a potential transformation into a concrete motor plan. We surmise that in our study, increased SMC and pSMA activation in the match condition reflects top-down integration processes of sublexical cues in the processing of DW2 (Tourville and Guenther 2011; Hertrich et al. 2016; Sohoglu and Davis 2020). Note that the cue for the verbal response was jittered in our experimental design. Therefore, we consider it unlikely that the activation differences in SMA and SMC reflect differences in speech preparation (Hervais-Adelman et al. 2012).

In line with a previous fMRI study investigating BOLD activation changes related to speech comprehension of degraded speech (Erb et al. 2013), we found activation increases in bilateral putamen in the match interaction contrast. This suggests a basal ganglia contribution to successfully degraded speech perception at sublexical processing. The basal ganglia seem important for temporal rhythm and temporal speech structure processing and may contribute to successful speech perception (Grahn and Rowe 2013; Kotz and Schmidt-Kassow 2015). However, the exact speech features driving basal ganglia are still unknown.

### PAC ROI analysis

Our ROI analysis in the Te1.0, Te1.1, and Te1.2 areas of PAC demonstrates that sublexical context modulates processing of degraded sublexical speech already at the earliest hierarchical levels of cortical auditory processing, indicating a top-down influence of speech context. In accordance with a neuroimaging study by Wild et al. (2012) investigating the modulatory effect of matching and mismatching visually presented sentences on degraded auditory sentence perception, we observed increased activation in area Te1.0 for the match condition compared with the mismatch condition. However, we observed greater responses to the match condition bilaterally in area Te1.0 and in areas Te1.1 and left Te1.2. Therefore, the overall sensitivity of the PAC to degraded speech seems to be increased by previous exposure to intact speech, even in the absence of semantic and syntactic information. The observed increase in PAC activation might reflect a rapidly induced plasticity to spectrotemporal stimulus features induced by CW presentation and might at least in part underlie successful perception. In electrophysiological recordings from PAC in ferrets, short-term plastic changes were observed with sharpened representations for task-relevant auditory stimuli (David et al. 2012; Yin et al. 2014). Yin et al. (2014) showed rapid spectrotemporal tuning in ferret PAC with the improved tuning of the recorded spectrotemporal fields to the spectrotemporal task-relevant features of the auditory stimuli. In a recent MEG study, speech envelope tracking was enhanced in PAC around 100 ms after degraded sentence onset when the sentence was intelligible due to prior clear sentence presentation (Di Liberto et al. 2018). These findings support our interpretation of the effects of sublexical cues in PAC for successful speech perception. Thus, spectrotemporal tuning in PAC and speech-specific tuning in STS/STG and possibly PAC may, to a large part, underlie the increased speech intelligibility of degraded sublexical cues observed in our study.

### Experimental and methodological considerations

The stronger activation increases, we found for DW2 after the presentation of a matching CW compared with a nonmatched CW in multiple areas of the cortical speech network, are in concordance with the results of Holdgraf et al. (2016). These authors recorded intracranially high gamma responses to acoustically presented lexico-semantic sentences in a comparable degraded-clear-degraded sequence. Similar to us, they found increased responses in multiple electrodes placed over the human cortex. High gamma responses are thought to be the main driver of the BOLD responses we analyzed in our study (Jensen et al. 2007; Scheeringa et al. 2011). It should be noted that other studies (Blank and Davis 2016; Sohoglu and Davis 2020) found decreased brain responses to degraded speech stimuli when it was preceded by a written matching clear word. However, there are numerous differences between these studies and ours. To name some, Sohoglu and Davis (2020) as well as Blank and Davis (2016) used written instead of acoustic words as cues and repeated the same degraded words multiple times, their analysis was not referenced to an initial presentation of the degraded speech stimulus, and the degraded stimuli were better recognizable than ours (Blank and Davis 2016). Nevertheless, the different effects of the clear cue on the brain activation elicited by degraded speech in the two groups of studies raise an important question regarding the generalizability of effects across different stimulation protocols that should be addressed in future studies. Using the same stimulation protocol with sublexical speech stimuli, our study replicated the enhanced activation after a matching cue reported by Holdgraf et al. (2016), who used lexico-semantic speech.

One challenge of neuroimaging studies is interpreting the results when using acoustic words with lexico-semantic information. It is unclear whether the increased brain activities are associated with the speech meaning and/or complex interaction of auditory representations. To avoid that conflict, we used auditorily presented sublexical pseudowords as speech stimuli. These pseudowords make up a proper word in a language but are not part of a person’s lexicon. Furthermore, we reduced the pseudowords intelligibility, i.e. degraded them, by applying a modulation filtering procedure (Elliott and Theunissen 2009; Santoro et al. 2014). Elliott and Theunissen (2009) concluded that the most important areas for speech intelligibility in the MPS are those with intermediate power. Therefore, we deleted those regions from the MPS to degrade pseudoword intelligibility.

### Potential limitations

We employed continuous instead of sparse EPI-sampling. Consequently, there was a continuous background noise from the scanner, which could have increased the difficulty of the listening task. In order to dampen the scanner noise, we used over-ear ear protectors and presented the stimuli with in-ear headphones that provide additional damping and are located underneath the ear protectors. At the behavioral level, the increase of pseudoword repetition accuracy in match trials indicates that the remaining background noise did not disturb the processing of the pseudowords sufficiently to suppress the effect of CW on DW2. Moreover, the background noise was the same for match and mismatch conditions. Therefore, the potential impact of the background noise on listening effort and brain processes should be matched between pseudowords and conditions. Importantly, background noise was also matched in the fMRI interaction analysis. Finally, if anything, excess levels of scanner background noise should have reduced the effects, we report at the brain and the behavioral level.

## Conclusion

Our results show that sublexical cues from prior clear pseudoword presentations improve degraded speech perception and activate the fronto-temporo-parietal network related to speech processing. This is evidence that several brain areas of the speech processing network, including posterior superior temporal cortex, AG, SMG, and SMC, are activated during the processing of meaningless sublexical speech stimuli. Furthermore, we found that the sublexical cues of clear speech preceding degraded speech affect the early hierarchical stage of cortical auditory processing in the PAC. These findings may be due to top-down effects on sensory processing due to sublexical cues.

## Supplementary Material

supplementary_materials_tgac007
