# Supplementary material for: Sublexical cues affect degraded speech processing: insights from fMRI
Source: Cereb Cortex Commun. 2022 Feb 16;3(1):tgac007. doi: 10.1093/texcom/tgac007 (PMC8914075; doi:10.1093/texcom/tgac007)
Supplement: supplementary_materials_tgac007 [file supplementary_materials_tgac007.zip › supplementary materials_SublexicalCues_Al-Zubaidi et al_proof.docx]

**Sublexical cues affect degraded speech processing: insights from fMRI**

Arkan Al-Zubaidi^1,2^, Susann Bräuer^1^, Chris R. Holdgraf^3^, Inga M. Schepers^1,2^ Jochem W. Rieger^1,2^

1. Applied Neurocognitive Psychology Lab and Cluster of Excellence Hearing4all, Oldenburg University, Oldenburg, Germany
2. Research Center Neurosensory Science, Oldenburg University, Oldenburg, Germany
3. Department of Statistics, UC Berkeley, California 94720, USA and International Interactive Computing Collaboration

*Correspondence should be sent to*

Prof. Jochem W. Rieger

Department of Psychology, Faculty VI

Oldenburg University

26129 Oldenburg

Germany

Tel: +49 (0)441 798 4533

E-mail: jochem.rieger@uni-oldenburg.de

**Behavioral results**

On average, in catch trials, participants repeated DWs more accurately in match trials (M = 56 %, SD = 15.3 %) than mismatch trials (M = 1 %, SD = 1.9 %; Figure S1). This difference was statistically significant in a paired t-test (t (19) = 18.1, p < 0.001). Furthermore, the percent of CW repeat was calculated for each mismatch trial. For the incorrect response to mismatch condition, the subjects reported 13.6% of what they heard in CW instead of DW (Figure S2).

| **Figure S1:** **Behavioral results of the speech intelligibility task of catch trials.** Matching clear in the catch trials pseudowords significantly increased the intelligibility of the degraded pseudowords in a paired t-test (*** represent p < 0.001). Bars show the mean with the standard deviation over subjects. | 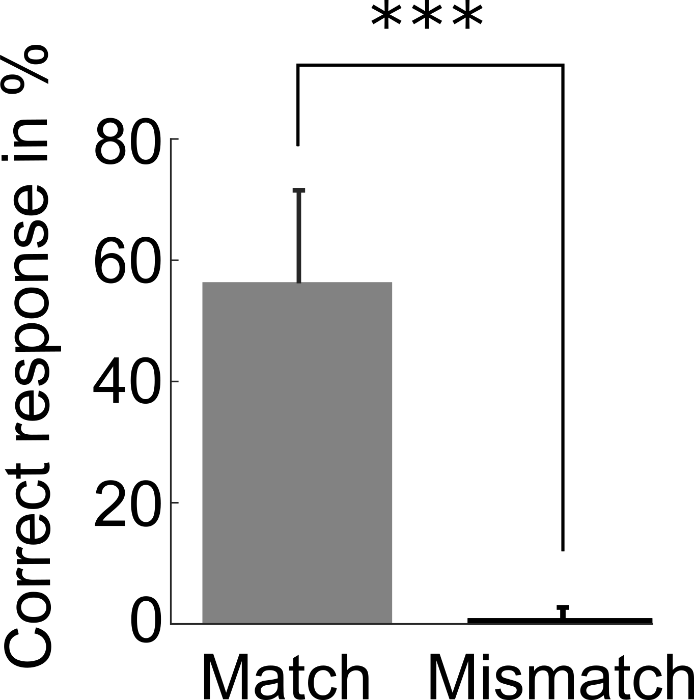 |
| --- | --- |

| **Figure S2: The average percentage of clear pseudoword repetitions over subjects in the mismatch trials.** Note that subjects adhered to the task instructions and reported the clear pseudoword in only 13.6% of the trials. | 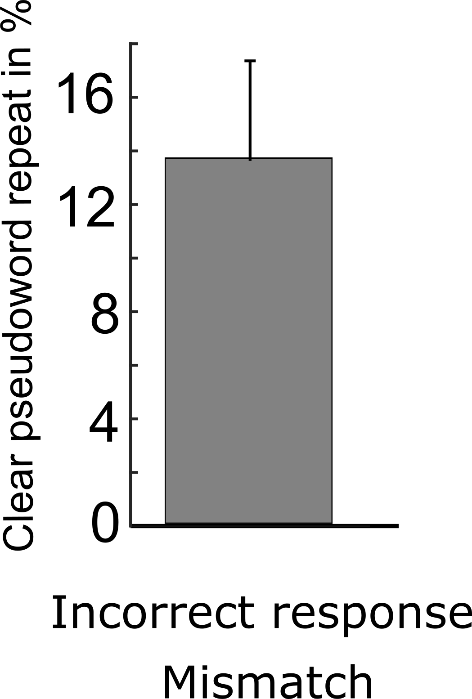 |
| --- | --- |
| **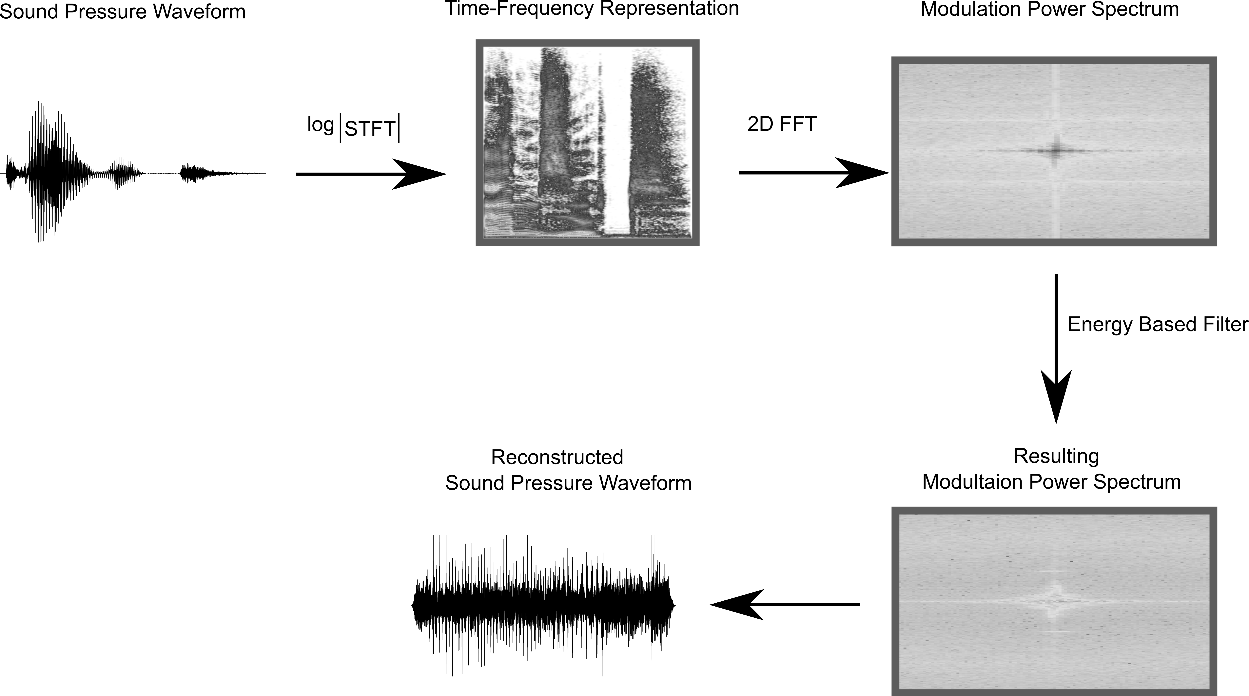** | |
| **Figure S3: Degraded speech stimulus preparation.** To reduce speech intelligibility the sound pressure waveform was first transformed to the log-spectrogram using the short-time Fourier transform. To obtain the modulation power spectrum (MPS) a 2D Fast Fourier transform (2D FFT) was applied to the spectrogram. The MPS was further modified by calculating the logarithmically-normalized energy distribution of the joint spectrotemporal modulations and zeroing all data points with a normalized energy between 0.7 and 0.75. The resulting MPS was then back-transformed into the log-spectrogram using the inverse 2D FFT and finally the filtered sound waveform was reconstructed via an inverse spectrogram algorithm. | |

**Primary auditory cortex ROI analysis**

We calculated the standard deviation (std) of beta-values corresponding to DW1 and DW2 for each subject, condition and ROI separately. We computed a 2x3x2 factorial rm-ANOVA with factors condition (match, mismatch), ROI (Te1.0, Te1.1, Te1.2) and hemisphere (left, right). There was no significant main effect for the condition (F (1,19)=1.2, p=0.4), hemisphere (F (1,19)= 0.09, p=0.8) or ROI (F (1,18)=1.1, p=0.5) and there were no interaction effects with the factor condition.

| 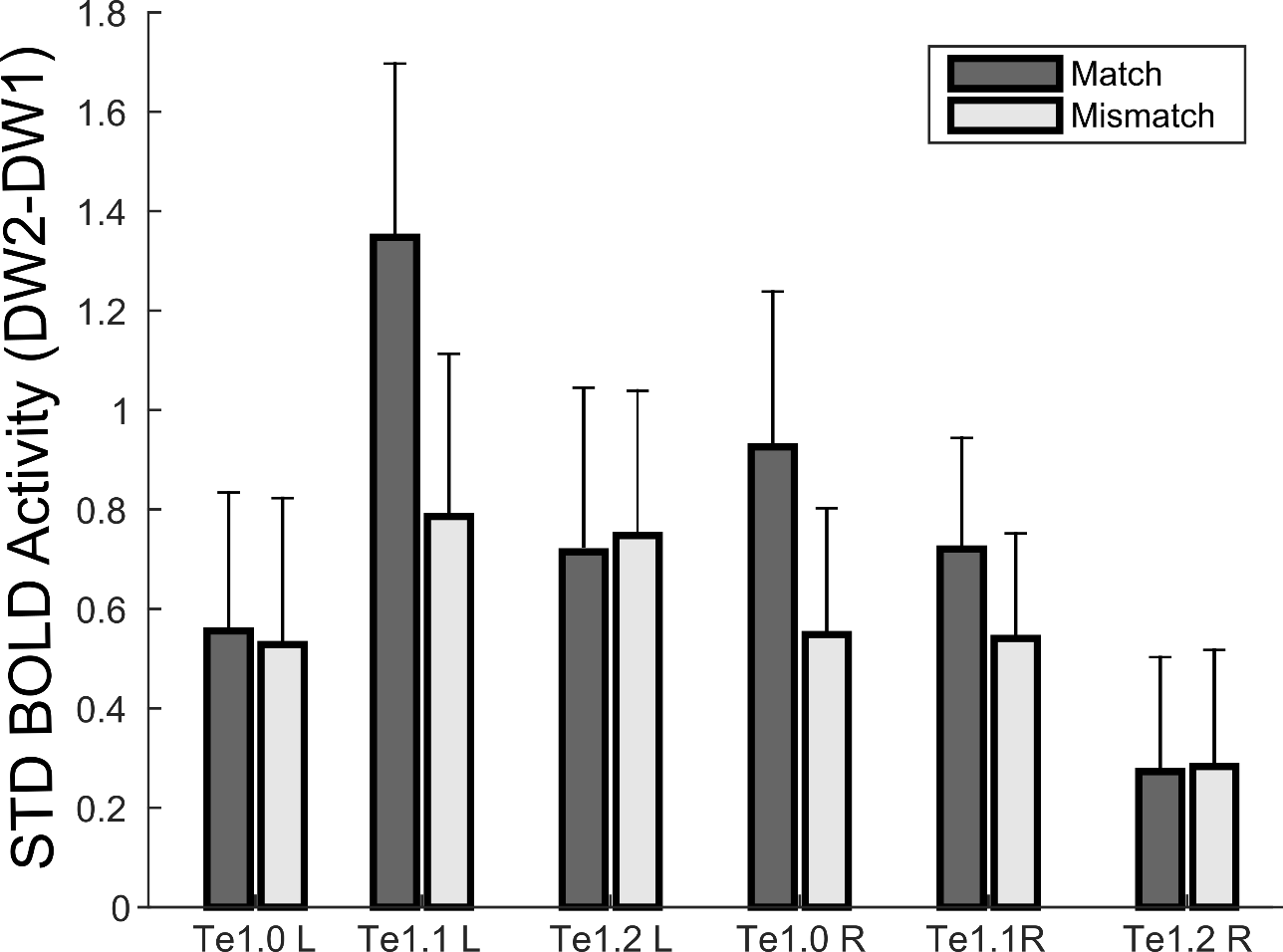 |
| --- |
| **Figure S4: Effects of matching clear pseudowords on degraded pseudoword on the standard deviation (std) of voxels in primary auditory cortex (PAC) regions of interest (ROIs).** We computed the rm-ANOVA on std calculated across voxels in each ROI. We found no indication for a std change. |

| 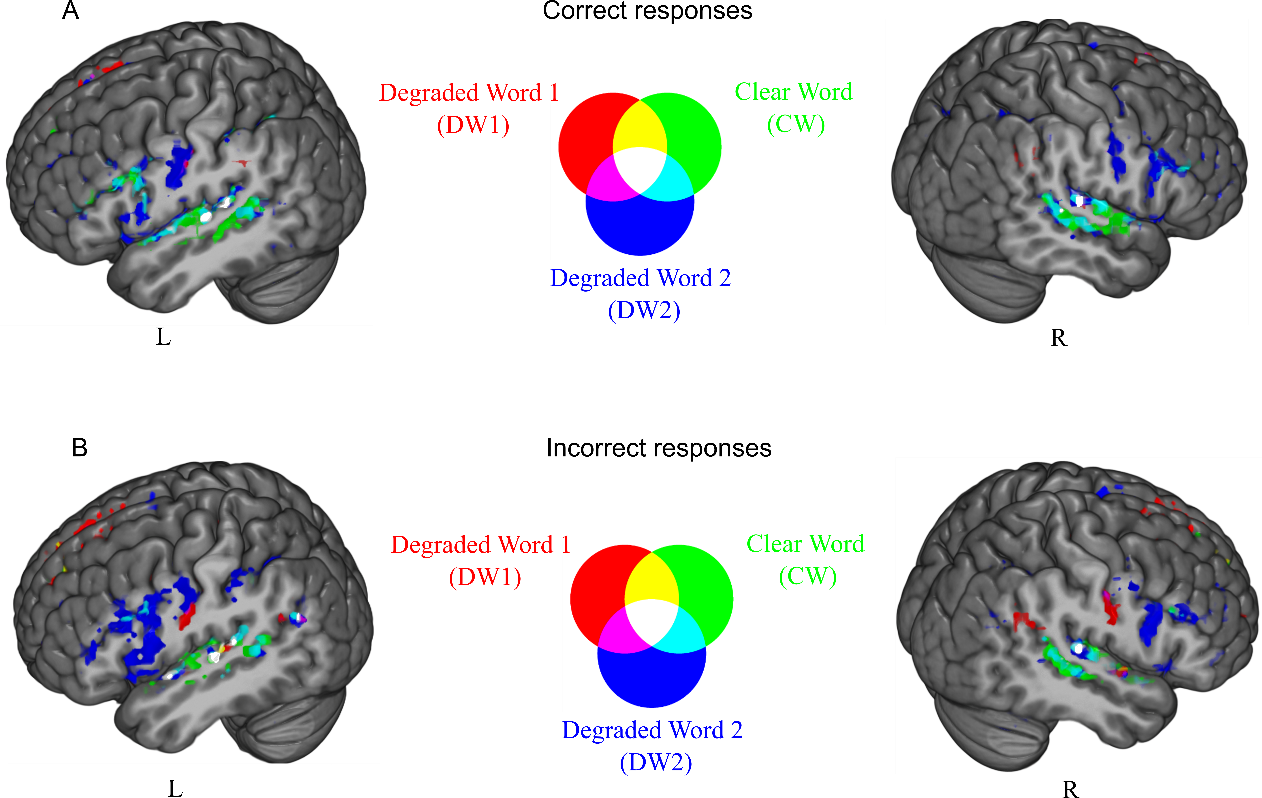 |
| --- |
| **Figure S5: FMRI BOLD activations elicited by clear and degraded pseudowords**. Clusters of brain activation related to correct (A) vs incorrect (B) identification of DW2. Results for the main effects of the degraded pseudoword at the beginning of each trial (DW1, red), the clear intermediate pseudoword (CW, green) and the subsequently presented degraded pseudoword (DW2, blue). Conjunction results of DW1 and CW (yellow), CW and DW2 (light blue), DW2 and DW1 (magenta) and all pseudoword types (white). All statistical results are shown for cluster-wise significance using a cluster defining threshold p < 0.001 (uncorrected) at the voxel level and p < 0.05 FEW corrected at the cluster level**.** |


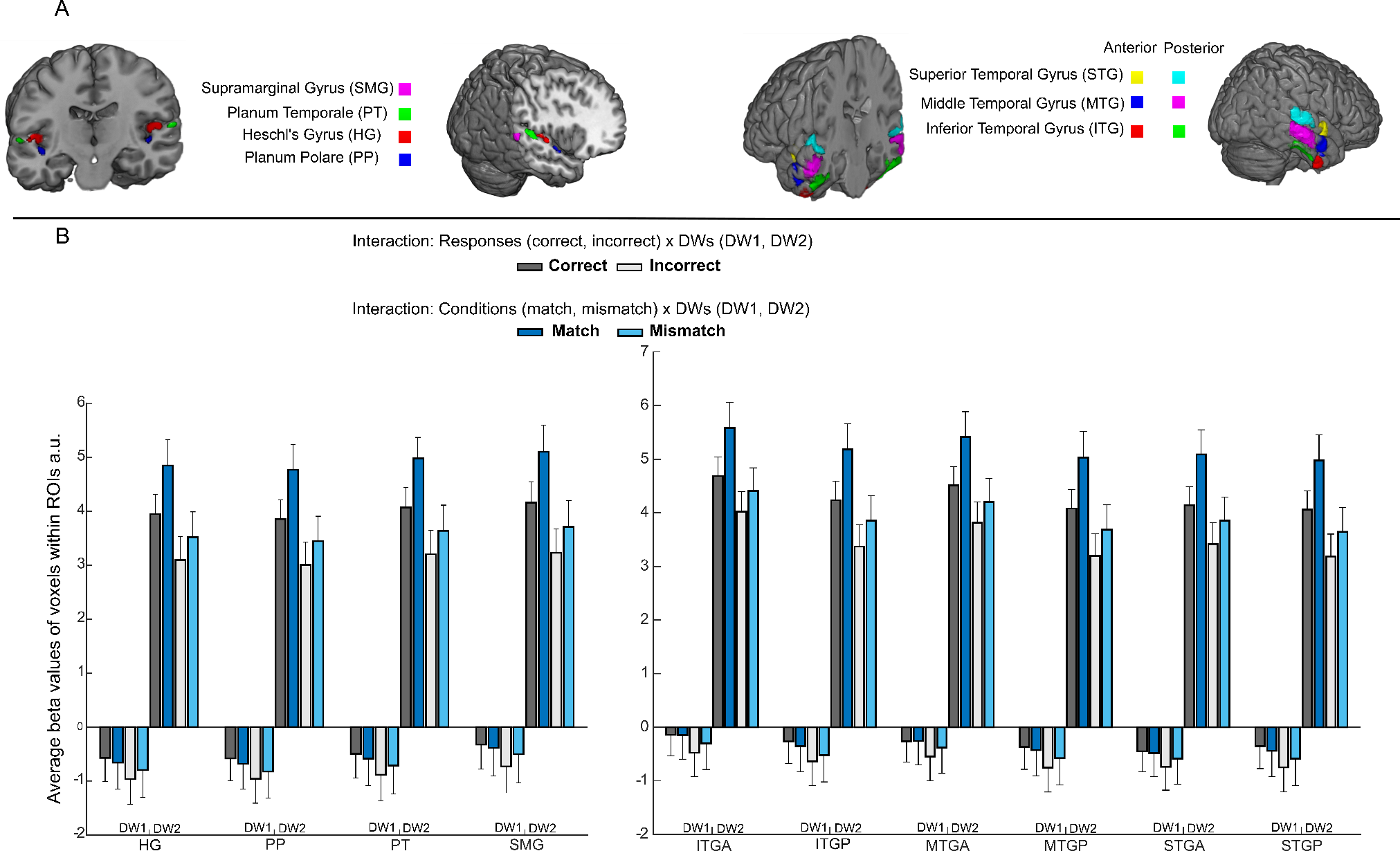


| **Figure S6: Average beta-values across voxels within ROIs.** (A) ROIs based on Harvard-Oxford cortical and subcortical Atlas. (B) Interactions between the response identification of DW2 (correct, incorrect) or type of clear pseudoword (matching, mismatching conditions) and position of degraded pseudowords (DW1, DW2) in different areas of the speech network. The beta-values indicate increased activation to DW2 for match trials (dark blue) compared to mismatch trials (light blue). For DW1, activation is similar across match/mismatch conditions. A similar pattern is observed when trials are sorted with respect to correct/incorrect responses. The DW2 response enhancement appears slightly weaker in the correct (dark grey) than the incorrect trials (light grey). Abbreviations: a.u, arbitrary units; HG, Heschl’s gyrus; PP, planum polare; PT, planum temporale; SMG, supramarginal gyrus; TGA, inferior temporal gyrus anterior; ITGP, inferior temporal gyrus posterior; MTGA, middle temporal gyrus anterior; MTGP, middle temporal gyrus posterior; STGA, superior temporal gyrus anterior; STGP, superior temporal gyrus posterior. |
| --- |

| Table S1: Summary of the one-way ANOVA and conjunction analyses findings the brain areas that activation changes in response to three pseudowords (DW1, CW and DW2) | | | | | | | |
| --- | --- | --- | --- | --- | --- | --- | --- |
| Regions | p(adj.) | K | F value | Local maxima (*x y z*) (mm) | | | Post-hoc t-tests |
| **Main effect: DW1** | | | | | | | |
| **Left superior temporal cortex** | **0.000** | **962** | **170** | **-53** | **-26** | **7** | **Activation > 0** |
| Left superior temporal cortex |  |  | 70 | -38 | -35 | 11 | Activation > 0 |
| **Right superior temporal cortex** | **0.000** | **928** | **141** | **63** | **-23** | **7** | **Activation > 0** |
| Right superior temporal cortex |  |  | 103 | 45 | -17 | 1 | Activation > 0 |
| **Left superior frontal medial gyrus** | **0.000** | **6702** | **78** | **-8** | **42** | **45** | **Activation < 0** |
| Left superior frontal gyrus |  |  | 72 | -14 | 34 | 57 | Activation < 0 |
| Left Anterior cingulate cortex |  |  | 52 | -2 | 25 | 17 | Activation < 0 |
| **Left inferior parietal gyrus** | **0.000** | **435** | **37** | **-56** | **-47** | **38** | **Activation < 0** |
| Left angular gyrus |  |  | 33 | -53 | -62 | 29 | Activation < 0 |
| Left middle occipital gyrus |  |  | 25 | -38 | -76 | 38 | Activation < 0 |
| **Right angular gyrus** | **0.000** | **334** | **35** | **54** | **-53** | **35** | **Activation < 0** |
| Right angular gyrus |  |  | 13 | 39 | -56 | 26 | Activation < 0 |
| **Left precentral gyrus** | **0.000** | **185** | **34** | **-32** | **-14** | **42** | **Activation < 0** |
| Left insuls cortex |  |  | 13 | -29 | -17 | 23 | Activation < 0 |
| **Right precentral gyrus** | **0.002** | **149** | **26** | **33** | **-14** | **38** | **Activation < 0** |
| Right postcentral gyrus |  |  | 15 | 57 | -5 | 23 | Activation < 0 |
|  | | | | | | | |
| **Main effect: CW** | | | | | | | |
| **Left middle temporal cortex** | **0.000** | **2962** | **355** | **-59** | **-38** | **11** | **Activation > 0** |
| Left superior temporal cortex |  |  | 229 | -53 | -8 | -2 | Activation > 0 |
| Left superior temporal cortex |  |  | 207 | -44 | -23 | 1 | Activation > 0 |
| **Right superior temporal cortex** | **0.000** | **2884** | **313** | **60** | **-20** | **1** | **Activation > 0** |
| Right superior temporal cortex |  |  | 234 | 57 | -2 | -8 | Activation > 0 |
| Right superior temporal cortex |  |  | 177 | 63 | -35 | 7 | Activation > 0 |
| **Right cerebelum 8** | **0.000** | **471** | **77** | **25** | **-67** | **-48** | **Activation > 0** |
| Right cerebelum crus1 |  |  | 48 | 10 | -76 | -27 | Activation > 0 |
| Right celebelum crus1 |  |  | 43 | 39 | -64 | -30 | Activation > 0 |
| **Left inferior temporal cortex** | **0.000** | **3132** | **76** | **-41** | **-41** | **-17** | **Activation > 0** |
| Left superior frontal gyrus |  |  | 74 | -14 | 48 | 35 | Activation < 0 |
| Left superior frontal gyrus |  |  | 56 | -14 | 34 | 57 | Activation < 0 |
| **Left inferior parietal gyrus** | **0.000** | **535** | **60** | **-41** | **-44** | **45** | **Activation > 0** |
| Left inferior parietal gyrus |  |  | 54 | -29 | -56 | 45 | Activation > 0 |
| Left inferior parietal gyrus |  |  | 14 | -50 | -26 | 48 | Activation > 0 |
| **Left supplementary motor area** | **0.000** | **236** | **57** | **-2** | **16** | **48** | **Activation > 0** |
| **Right middle cingulate cortex** | **0.000** | **229** | **39** | **16** | **-2** | **29** | **Activation < 0** |
| Right superior frontal gyrus |  |  | 15 | 30 | -11 | 32 | Activation < 0 |
| **Right inferior parietal gyrus** | **0.000** | **326** | **37** | **36** | **-47** | **45** | **Activation > 0** |
| Right postcentral gyrus |  |  | 18 | 45 | -26 | 51 | Activation > 0 |
| **Left cuneus cortex** | **0.000** | **1312** | **36** | **-8** | **-79** | **17** | **Activation > 0** |
| Right calcarine cortex |  |  | 32 | 13 | -70 | 17 | Activation > 0 |
| Right calcarine cortex |  |  | 31 | 7 | -82 | 7 | Activation > 0 |
| **Right middle temporal cortex** | **0.018** | **91** | **35** | **54** | **-5** | **-27** | **Activation < 0** |
| Right middle temporal pole |  |  | 29 | 48 | 13 | -33 | Activation < 0 |
| Right inferior temporal cortex |  |  | 13 | 57 | -20 | -20 | Activation < 0 |
| **Left angular gyrus** | **0.003** | **136** | **34** | **-47** | **-70** | **32** | **Activation < 0** |
| **Left posterior cingulate cortex** | **0.003** | **139** | **25** | **-2** | **-44** | **32** | **Activation < 0** |
| Left precuneus |  |  | 23 | -8 | -56 | 23 | Activation < 0 |
| **Left putamen** | **0.031** | **79** | **19** | **-11** | **10** | **-2** | **Activation > 0** |
|  | | | | | | | |
| **Main effect: DW2** | | | | | | | |
| **Left middle temporal cortex** | **0.000** | **33493** | **376** | **-59** | **-38** | **11** | **Activation > 0** |
| Left supplementary motor area |  |  | 309 | -2 | 16 | 45 | Activation > 0 |
| Right superior temporal cortex |  |  | 297 | 63 | -20 | 1 | Activation > 0 |
| Left superior temporal cortex |  |  | 289 | -44 | -20 | -2 | Activation > 0 |
| Right superior temporal cortex |  |  | 280 | 45 | -20 | -2 | Activation > 0 |
| Left supplementary motor area |  |  | 268 | -2 | 7 | 60 | Activation > 0 |
| Right cerebelum 8 |  |  | 245 | 22 | -67 | -48 | Activation > 0 |
| Left cerebelum crus1 |  |  | 231 | -8 | -76 | -27 | Activation > 0 |
| Right cerebelum crus1 |  |  | 231 | 36 | -67 | -30 | Activation > 0 |
| Right insula cortex |  |  | 229 | 33 | 25 | -2 | Activation > 0 |
| Left inferior frontal operculum |  |  | 217 | -53 | 10 | 14 | Activation > 0 |
| Left cerebelum 7 |  |  | 216 | -26 | -73 | -48 | Activation > 0 |
| Left precentral gyrus |  |  | 210 | -47 | 4 | 29 | Activation > 0 |
| Left insula cortex |  |  | 203 | -28 | 22 | 1 | Activation > 0 |
| Left superior temporal cortex |  |  | 186 | -38 | -38 | 11 | Activation > 0 |
| **Left middle occipital gyrus** | **0.000** | **277** | **82** | **-41** | **-76** | **38** | **Activation < 0** |
| **Left orbital frontal medial gyrus** | **0.000** | **351** | **47** | **-2** | **57** | **-8** | **Activation < 0** |
| Left orbital frontal medial gyrus |  |  | 43 | -2 | 37 | -11 | Activation < 0 |
| **Left middle temporal cortex** | **0.03** | **77** | **30** | **-56** | **-14** | **-17** | **Activation < 0** |
| Left middle temporal cortex |  |  | 21 | -53 | 4 | -27 | Activation < 0 |
| **Left middle cingulate cortex** | **0.003** | **134** | **29** | **-2** | **-41** | **35** | **Activation < 0** |
| Left precuneus |  |  | 29 | -11 | -56 | 17 | Activation < 0 |
|  |  |  |  |  |  |  |  |
| **Conjunction analysis: DW1 & CW** | | | | | | | |
| **Left superior temporal cortex** | **0.000** | **962** | **170** | **-53** | **-26** | **7** | **Activation > 0** |
| Left superior temporal cortex |  |  | 70 | -38 | -35 | 11 | Activation > 0 |
| **Right superior temporal cortex** | **0.000** | **927** | **141** | **63** | **-23** | **7** | **Activation > 0** |
| Right superior temporal cortex |  |  | 102 | 45 | -17 | 1 | Activation > 0 |
| **Left superior frontal gyrus** | **0.000** | **1462** | **70** | **-14** | **48** | **35** | **Activation < 0** |
| Left superior frontal gyrus |  |  | 56 | -14 | 34 | 57 | Activation < 0 |
| Left superior frontal medial gyrus |  |  | 43 | 1 | 51 | 26 | Activation < 0 |
| **Right middle temporal cortex** | **0.02** | **86** | **31** | **54** | **-5** | **-27** | **Activation < 0** |
| Right middle temporal pole |  |  | 29 | 48 | 13 | -33 | Activation < 0 |
| Right inferior temporal cortex |  |  | 13 | 57 | -20 | -20 | Activation < 0 |
| **Left angular gyrus** | **0.003** | **134** | **27** | **-50** | **-64** | **29** | **Activation < 0** |
| Left middle occipital gyrus |  |  | 25 | -38 | -76 | 38 | Activation < 0 |
|  |  |  |  |  |  |  |  |
| **Conjunction analysis: DW1 & DW2** | | | | | | | |
| **Left superior temporal cortex** | **0.000** | **962** | **170** | **-53** | **-26** | **7** | **Activation > 0** |
| Left superior temporal cortex |  |  | 70 | -38 | -35 | 11 | Activation > 0 |
| **Right superior temporal cortex** | **0.000** | **928** | 141 | **63** | **-23** | **7** | **Activation > 0** |
| Right superior temporal cortex |  |  |  | 45 | -17 | 1 | Activation > 0 |
| **Right superior frontal medial gyrus** | **0.000** | **890** | **44** | **4** | **39** | **38** | **Activation < 0 & > 0** |
| Right supplementary motor area |  |  | 37 | 1 | 22 | 63 | Activation < 0 & > 0 |
| Left Anterior cingulate cortex |  |  | 31 | -5 | 25 | 20 | Activation < 0 & > 0 |
| **Right orbital frontal inferior gyrus** | **0.000** | **526** | **33** | **36** | **22** | **-14** | **Activation < 0 & > 0** |
| Right orbital frontal middle gyrus |  |  | 29 | 36 | 51 | -5 | Activation < 0 & > 0 |
| Right putamen |  |  | 22 | 30 | -2 | -5 | Activation < 0 & > 0 |
| **Left angular gyrus** | **0.000** | **203** | **31** | **-53** | **-64** | **29** | **Activation < 0** |
| Left middle occipital gyrus |  |  | 25 | -38 | -76 | 38 | Activation < 0 |
| **Left middle frontal gyrus** | **0.000** | **532** | **31** | **-38** | **57** | **1** | **Activation < 0 & > 0** |
| Left middle frontal gyrus |  |  | 29 | -32 | 39 | 26 | Activation < 0 & > 0 |
| Left middle frontal gyrus |  |  | 27 | -26 | 45 | 1 | Activation < 0 & > 0 |
| **Left postcentral gyrus** | **0.003** | **133** | **30** | **-38** | **-14** | **42** | **Activation < 0 & > 0** |
| **Left orbital frontal medial gyrus** | **0.004** | **127** | **26** | **-1** | **57** | **-14** | **Activation < 0** |
| Left orbital frontal medial gyrus |  |  | 21 | -1 | 39 | -8 | Activation < 0 |
| Left Anterior cingulate cortex |  |  | 19 | -5 | 51 | 1 | Activation < 0 |
| **Right precentral gyrus** | **0.005** | **120** | **25** | **45** | **-11** | **32** | **Activation < 0 & > 0** |
| Right precentral gyrus |  |  | 15 | 57 | -5 | 23 | Activation < 0 & > 0 |
| **Right caudate** | **0.000** | **385** | **24** | **13** | **4** | **17** | **Activation < 0 & > 0** |
| Left insula cortex |  |  | 22 | -29 | 13 | -14 | Activation < 0 & > 0 |
| Left putamen |  |  | 21 | -26 | -11 | 1 | Activation < 0 & > 0 |
|  |  |  |  |  |  |  |  |
| **Conjunction analysis: CW & DW2** | | | | | | | |
| **Left middle temporal cortex** | **0.000** | **2931** | **355** | **-59** | **-38** | **11** | **Activation > 0** |
| Left superior temporal cortex |  |  | 207 | -44 | -23 | 1 | Activation > 0 |
| Left superior temporal cortex |  |  | 107 | -38 | -35 | 11 | Activation > 0 |
| **Right superior temporal cortex** | **0.000** | **2787** | **297** | **63** | **-20** | **1** | **Activation > 0** |
| Right superior temporal cortex |  |  | 173 | 63 | -35 | 7 | Activation > 0 |
| Right superior temporal cortex |  |  | 161 | 54 | -5 | -2 | Activation > 0 |
| **Right cerebelum 8** | **0.000** | **471** | **77** | **25** | **-67** | **-48** | **Activation > 0** |
| Right cerebelum crus1 |  |  | 48 | 10 | -76 | -27 | Activation > 0 |
| Right celebelum crus1 |  |  | 43 | 39 | -64 | -30 | Activation > 0 |
| **Left inferior temporal cortex** | **0.000** | **636** | **69** | **-44** | **-41** | **-17** | **Activation > 0** |
| Left cerebelum crus2 |  |  | 52 | -11 | -76 | -39 | Activation > 0 |
| Left celebelum crus1 |  |  | 40 | -38 | -59 | -33 | Activation > 0 |
| **Left inferior parietal gyrus** | **0.000** | **531** | **60** | **-41** | **-44** | **45** | **Activation > 0** |
| Left inferior parietal gyrus |  |  | 54 | -29 | -56 | 45 | Activation > 0 |
| Left inferior parietal gyrus |  |  | 14 | -50 | -26 | 48 | Activation > 0 |
| **Left supplementary motor area** | **0.000** | **236** | **57** | **-2** | **16** | **48** | **Activation > 0** |
| **Left orbital frontal medial gyrus** | **0.000** | **286** | **38** | **-2** | **39** | **-8** | **Activation < 0** |
| **Right inferior parietal gyrus** | **0.000** | **326** | **37** | **36** | **-47** | **45** | **Activation > 0** |
| Right postcentral gyrus |  |  | 19 | 45 | -26 | 51 | Activation > 0 |
| **Left cuneus cortex** | **0.000** | **1308** | **36** | **-8** | **-79** | **17** | **Activation > 0** |
| Right calcarine cortex |  |  | 32 | 13 | -70 | 17 | Activation > 0 |
| Right calcarine cortex |  |  | 31 | 7 | -82 | 7 | Activation > 0 |
| **Left angular gyrus** | **0.003** | **136** | **34** | **-47** | **-70** | **32** | **Activation < 0** |
| **Left posterior cingulate cortex** | **0.009** | **91** | **24** | **-2** | **-41** | **35** | **Activation < 0** |
| Left precuneus |  |  | 23 | -8 | -56 | 23 | **Activation < 0** |
| **Left putamen** | **0.03** | **78** | **19** | **-11** | **10** | **-2** | **Activation > 0** |
|  |  |  |  |  |  |  |  |
| **Conjunction analysis: DW1 & CW & DW2** | | | | | | | |
| **Left superior temporal cortex** | **0.000** | **962** | **170** | **-53** | **-26** | **7** | **Activation > 0** |
| Left superior temporal cortex |  |  | 70 | -38 | -35 | 11 | Activation > 0 |
| **Right superior temporal cortex** | **0.000** | **927** | **141** | **63** | **-23** | **7** | **Activation > 0** |
| Right superior temporal cortex |  |  | 103 | 45 | -17 | 1 | Activation > 0 |
| **Left angular gyrus** | **0.003** | **134** | **27** | **-50** | **-64** | **29** | **Activation < 0** |
| Left middle occipital gyrus |  |  | 25 | -38 | -76 | 38 | Activation < 0 |
| **Left orbital frontal medial gyrus** | **0.004** | **127** | **26** | **-1** | **57** | **-14** | **Activation < 0** |
| Left orbital frontal medial gyrus |  |  | 21 | -1 | 39 | -8 | Activation < 0 |
| Left Anterior cingulate cortex |  |  | 19 | -5 | 51 | 1 | Activation < 0 |
| Notes: The table shows three local maxima (MNI coordinates) more than 16.0 mm, the adjusted (adj.) p-values are reported at p<0.001 (height threshold) and p<0.05 (RWE extent threshold). F=peak of F values. K=cluster size. | | | | | | | |

| Table S2: Interaction between type of clear pseudoword (matching and mismatching conditions) and position of degraded pseudowords | | | | | | |
| --- | --- | --- | --- | --- | --- | --- |
| Regions | p(adj.) | K | F value | Local maxima (*x y z*) (mm) | | |
|  | | | | | | |
| **Right hippocampus** | **0.000** | **2043** | **38** | **33** | **-20** | **-8** |
| Right superior temporal gyrus and sulcus |  |  | 34 | 36 | -31 | 13 |
| Right supramarginal gyrus |  |  | 33 | 48 | -41 | 38 |
| Right inferior temporal gyrus |  |  | 32 | 51 | -44 | -11 |
| Right middle temporal gyrus |  |  | 33 | 57 | -53 | 7 |
| Right rolandic operculum |  |  | 29 | 51 | -8 | 14 |
| Right putamen |  |  | 28 | 33 | 4 | 4 |
| Right supramarginal gyrus |  |  | 27 | 57 | -32 | 32 |
| Right inferior frontal gyrus pars opercularis |  |  | 24 | 60 | 8 | 3 |
| Right somato-motor cortex |  |  | 21 | 56 | -31 | 44 |
| Right parahippocampal gyrus |  |  | 18 | 36 | -23 | -20 |
| Right Heschl gyrus |  |  | 15 | 42 | -20 | 11 |
| Right superior temporal cortex |  |  | 14 | 57 | -23 | 17 |
|  |  |  |  |  |  |  |
| **Left insular cortex** | **0.000** | **536** | **37** | **-35** | **-11** | **23** |
| Left somato-motor cortex |  |  | 32 | -43 | -23 | 45 |
| Left putamen |  |  | 28 | -26 | -8 | -8 |
| Left middle cingulate cortex |  |  | 21 | -2 | 1 | 45 |
| Left supplementary motor area |  |  | 20 | -1 | -12 | 55 |
| Left inferior frontal gyrus pars opercularis |  |  | 17 | -47 | 5 | 14 |
|  |  |  |  |  |  |  |
| **Left angular gyrus** | **0.000** | **306** | **26** | **-44** | **-53** | **26** |
| Left inferior parietal gyrus |  |  | 23 | -45 | -61 | 23 |
| Notes: The table shows ten local maxima (MNI coordinates) more than 16.0 mm, the adjusted (adj.) p-values are reported at p<0.001 (height threshold) and p<0.05 (RWE extent threshold). F=peak of F values. K=cluster size. | | | | | | |

| Table S3: rm-ANOVA and post hoc comparisons of average beta values of voxels within ROIs | | | | | | | |
| --- | --- | --- | --- | --- | --- | --- | --- |
| ROIs | rm-ANOVA: F (1,19), Significance (p-value) | | | Paired t-test with Bonferroni’s correction (alpha=0.05): Mean difference, Significance (p-value) | | | |
|  | Main effect condition (match vs. mismatch) | Main effect degraded pseudowords (DW1 vs. DW2) | Interaction condition x pseudowords | DW1 (match vs. mismatch) | DW2 (match vs. mismatch) | Match (DW1 vs. DW2) | Mismatch (DW1 vs. DW2) |
| SMG | F = 14.5* | F = 54*** | F = 17** | MD = 0.11^ns^ | MD = 1.4** | MD = -5,5** | MD = -4.1** |
| PP | F = 15.5* | F = 60*** | F = 18.3** | MD = 0.14^ns^ | MD = 1.3 ** | MD = -5.4** | MD = -4.2** |
| HG | F = 10.8* | F = 56*** | F = 17.4** | MD = 0.13^ns^ | MD = 1.3** | MD = -5.5** | MD = -4.3** |
| PT | F = 14.5* | F = 56*** | F = 17** | MD = 0.13^ns^ | MD = 1.3** | MD = -5.6** | MD = -4.4** |
| STGA | F = 13.3* | F = 73*** | F = 19** | MD = 0.19^ns^ | MD = 1.2** | MD = -5.5** | MD = -4.4** |
| STGP | F = 15.3** | F = 60*** | F = 17.7** | MD = 0.2^ns^ | MD = 1.3** | MD = -5.4** | MD = -4,2** |
| MTGA | F = 12.3* | F = 79*** | F = 16** | MD = 0.12^ns^ | MD = 1.2** | MD = -5.6** | MD = -4.5** |
| MTGP | F = 15.4** | F = 61*** | F = 17.7** | MD = 0.2^ns^ | MD = 1.3** | MD = -5.4** | MD = -4.3** |
| ITGA | F = 11.9* | F = 85*** | F = 13** | MD = 0.15^ns^ | MD = 1.2** | MD = -5.7** | MD = - 4.7** |
| ITGP | F = 14.7* | F = 65*** | F = 17** | MD = 0.16^ns^ | MD = 1.3** | MD = -5.5** | MD = -4.4** |
| MD: Mean Difference; ns = not significant; * = p<0.005; ** = p<0.001; *** = p<0.00001 | | | | | | | |
