# Supplementary figures and images for: Sublexical cues affect degraded speech processing: insights from fMRI
Source: Cereb Cortex Commun. 2022 Feb 16;3(1):tgac007. doi: 10.1093/texcom/tgac007 (PMC8914075; doi:10.1093/texcom/tgac007)

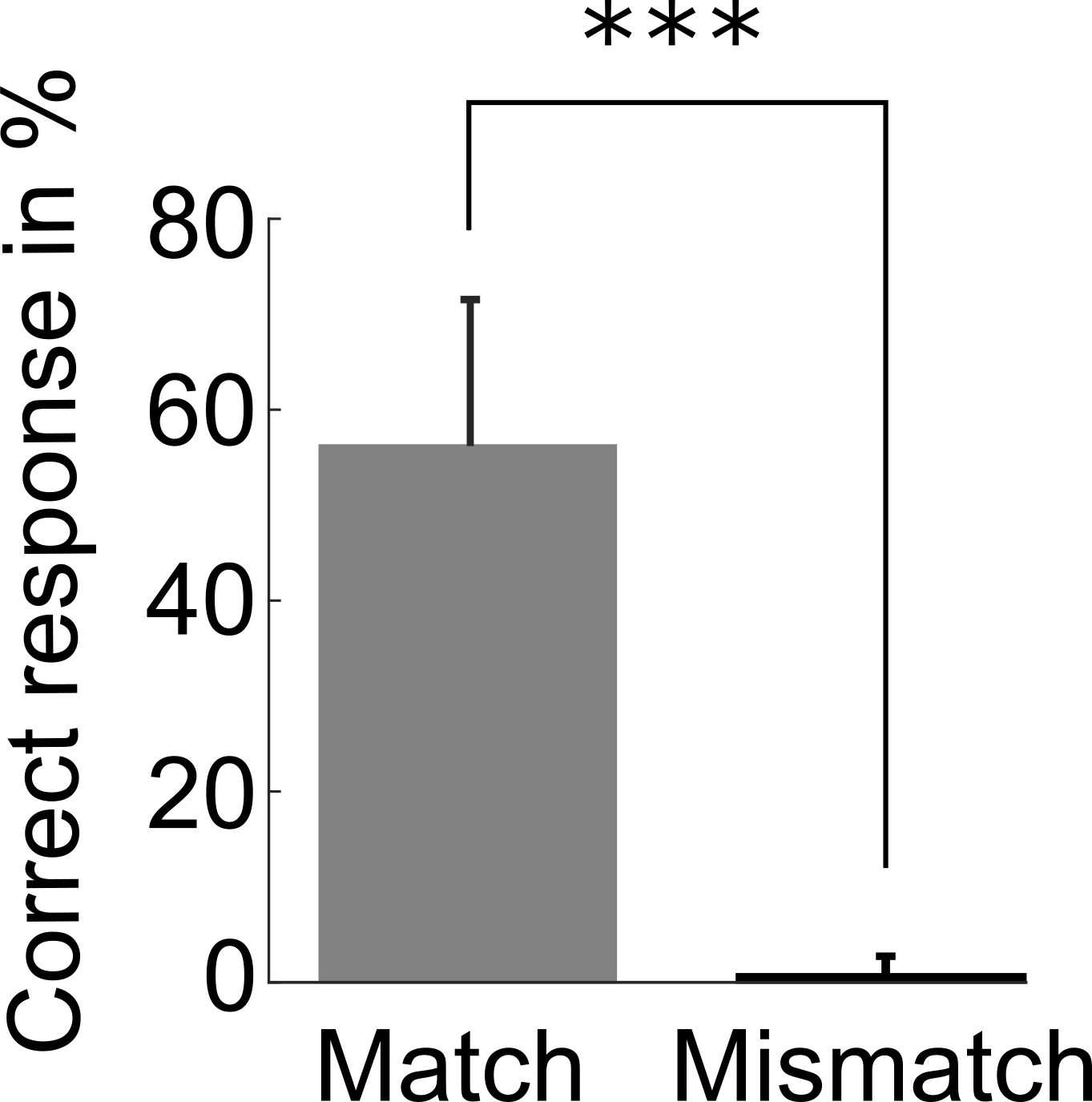

Supplement: supplementary_materials_tgac007 [file supplementary_materials_tgac007.zip › S1_Catch.tif]

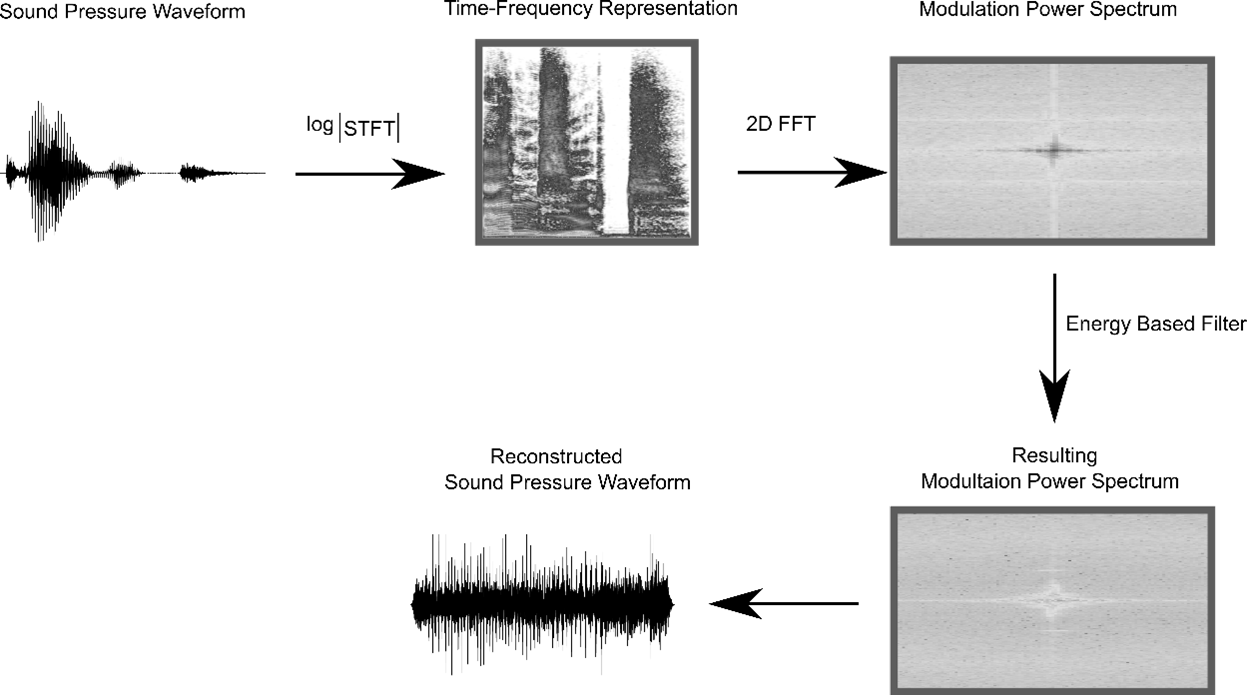

Supplement: supplementary_materials_tgac007 [file supplementary_materials_tgac007.zip › S3.tif]

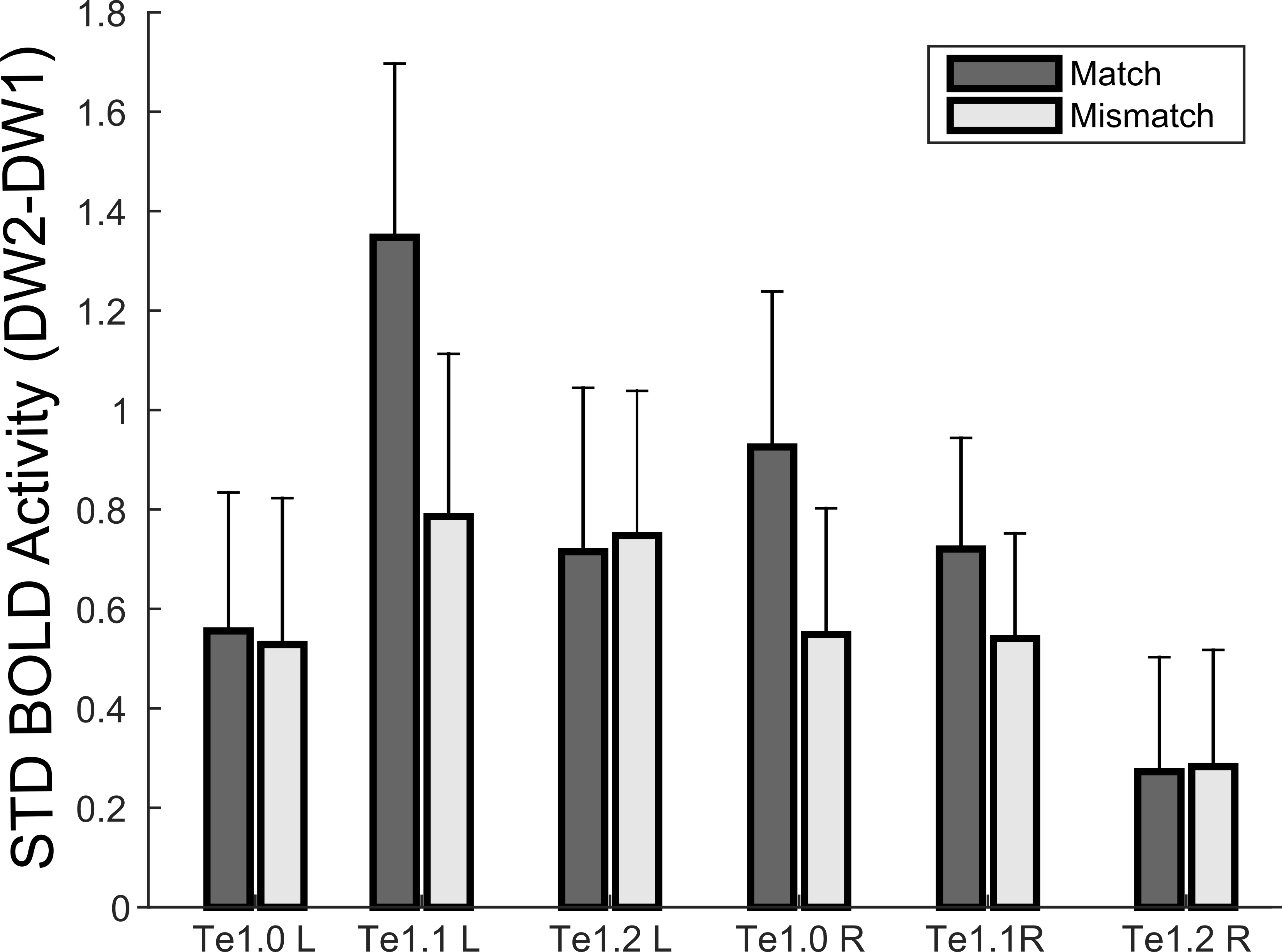

Supplement: supplementary_materials_tgac007 [file supplementary_materials_tgac007.zip › S4_STD_ROI_PAC.tif]

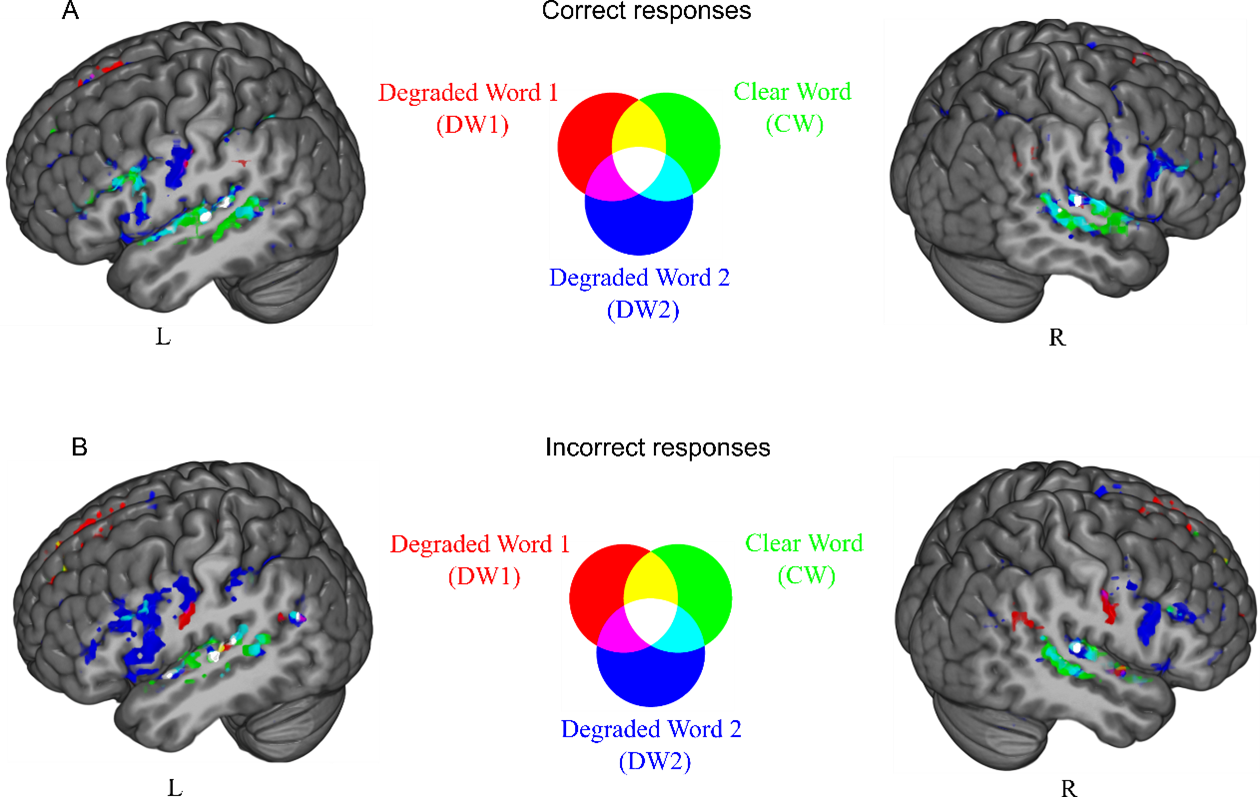

Supplement: supplementary_materials_tgac007 [file supplementary_materials_tgac007.zip › S5.tif]

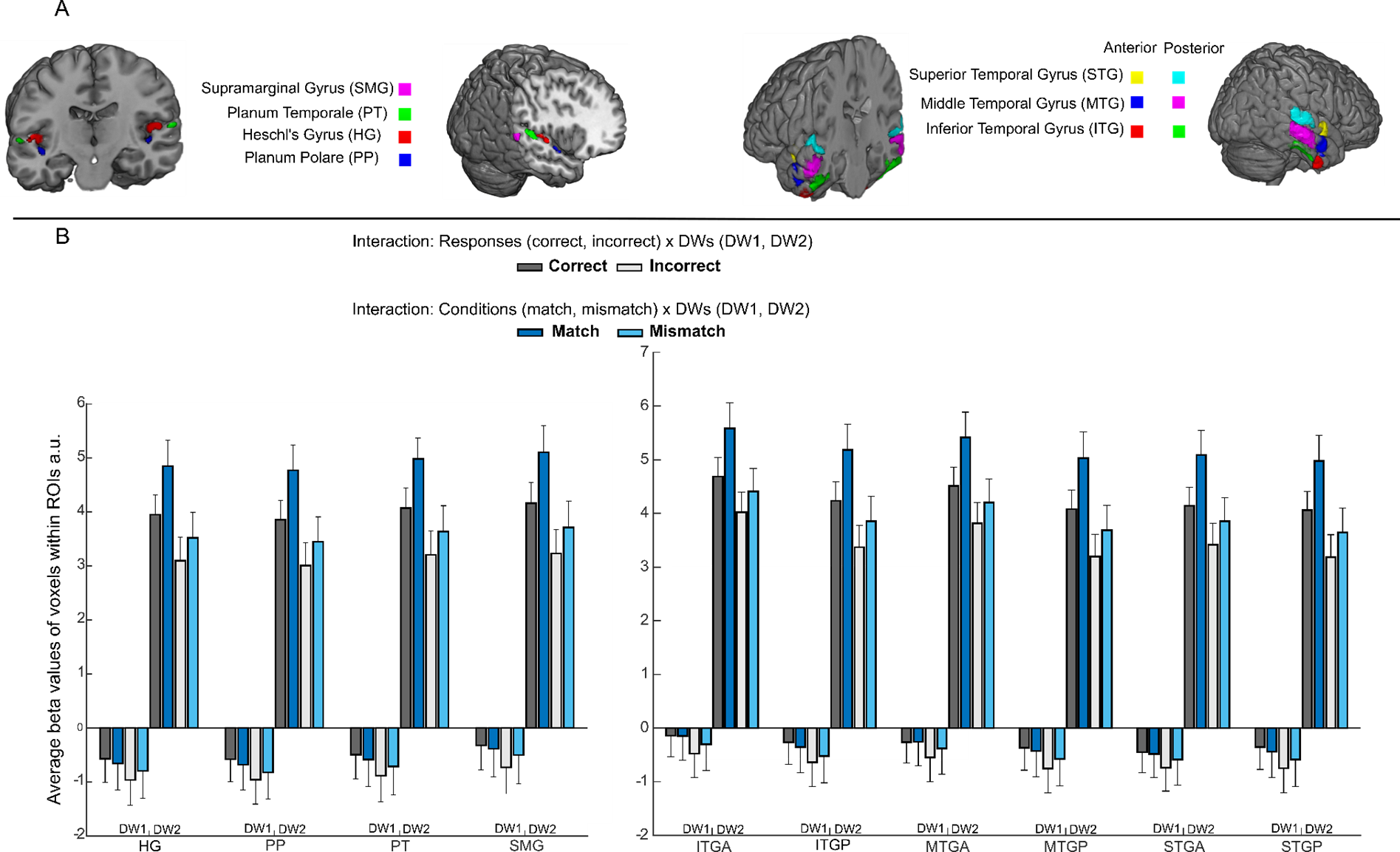

Supplement: supplementary_materials_tgac007 [file supplementary_materials_tgac007.zip › S6.tif]
